# Effect of molybdenum supply on crop performance through rhizosphere soil microbial diversity and metabolite variation

**DOI:** 10.3389/fpls.2024.1519540

**Published:** 2025-01-28

**Authors:** Muhammad Shoaib Rana, Dikhnah Alshehri, Rui-Long Wang, Muhammad Imran, Yousif Abdelrahman Yousif Abdellah, Faiz Ur Rahman, Marfat Alatawy, Hanaa Ghabban, Amany H. A. Abeed, Cheng-xiao Hu

**Affiliations:** ^1^ Guangdong Engineering Technology Research Centre of Modern Eco-agriculture and Circular Agriculture, Department of Ecology, College of Natural Resources and Environment, South China Agricultural University, Guangzhou, China; ^2^ Key Laboratory of Arable Land Conservation (Middle and Lower Reaches of Yangtze River), Ministry of Agriculture, Micro-elements Research Center, College of Resource and Environment, Huazhong Agricultural University, Wuhan, China; ^3^ Department of Biology, Faculty of Science, University of Tabuk, Tabuk, Saudi Arabia; ^4^ Biodiversity Genomics Unit, Faculty of Science, University of Tabuk, Tabuk, Saudi Arabia; ^5^ College of Agriculture, South China Agricultural University, Guangzhou, China; ^6^ The Germplasm Bank of Wild Species, Yunnan Key Laboratory for Fungal Diversity and Green Development, Kunming Institute of Botany, Chinese Academy of Sciences, Kunming, Yunnan, China; ^7^ Guangdong Provincial Key Laboratory of Postharvest Science of Fruits and Vegetables/Engineering Research Center for Postharvest Technology of Horticultural Crops in South China, Ministry of Education, College of Horticulture, South China Agricultural University, Guangzhou, Guangdong, China; ^8^ Department of Botany and Microbiology, Faculty of Science, Assiut Universityt, Assiu, Egypt

**Keywords:** maize, soybean, microbial diversity, metabolite variations, nutrient acquisition

## Abstract

Molybdenum (Mo) deficiency is a global problem in acidic soils, limiting plant growth, development, and nutrient availability. To address this, we carried out a field study with two treatments, i.e., Mo applied (+Mo) and without Mo (−Mo) treatment to explore the effects of Mo application on crop growth and development, microbial diversity, and metabolite variations in maize and soybean cropping systems. Our results indicated that the nutrient availability (N, P, K) was higher under Mo supply leading to improved biological yield and nutrient uptake efficiency in both crops. Microbial community analysis revealed that *Proteobacteria* and *Acidobacteria* were the dominant phyla in Mo treated (+Mo) soils for both maize and soybean. Both these phyla accounted together 39.43% and 57.74% in −Mo and +Mo, respectively, in soybean rhizosphere soil, while they accounted for 44.51% and 46.64% in maize rhizosphere soil. This indicates more variations among the treatments in soybean soil compared to maize soil. At a lower taxonomic level, the diverse responses of the genera indicated the specific bacterial community adaptations to fertilization. *Candidatus Koribacter* and *Kaistobacter* were commonly significantly higher in both crops under Mo-applied conditions in both cropping systems. These taxa, sharing similar functions, could serve as potential markers for nutrient availability and soil fertility. Metabolite profiling revealed 8 and 10 significantly differential metabolites in maize and soybean, respectively, under +Mo treatment, highlighting the critical role of Mo in metabolite variation. Overall, these findings emphasize the importance of Mo in shaping soil microbial diversity by altering metabolite composition, which in turn may enhance the nutrient availability, nutrient uptake, and plant performance.

## 1 Introduction

Molybdenum is essentially required by plants and rhizosphere microbiota for sustainable crop production. Mo deficiency is common in the acidic soils of China. Mo-deficient plants exhibit stunted growth and poor nutrient interaction is rhizosphere soil (Nie et al., 2015). Mo is an essential component of several enzymes, including nitrate reductase (NR), aldehyde oxidase (AO), and xanthine dehydrogenase (XDH), which play critical roles in the proper physiological functioning of various crops (Huang et al., 2022). Under Mo-deficient conditions, plants experience a variety of phenotypic changes that hinder their growth. These effects are primarily associated with the impaired activity of molybdoenzymes. Notably, these include key enzymes involved in N metabolism, such as NR and nitrogenase. Nitrogenase, in particular, plays a critical role in N fixation within the bacteroids of legume nodules. Other molybdoenzymes found in plants include XDH, which is essential for purine degradation and ureide synthesis in legumes; AO, which participates in the biosynthesis of abscisic acid; and sulfite oxidase, which converts sulfite to sulfate, a vital step in the metabolism of sulfur-containing amino acids (Mendel and Hänsch, 2002; Sauer and Frebort, 2003; Mendel and Kruse, 2012). The availability of Mo for plant uptake is significantly affected by soil pH, the presence of adsorbing oxides, such as iron oxides, water drainage conditions, and organic compounds in soil colloids. In alkaline soils with high pH, Mo becomes more soluble and is predominantly available to plants in its anionic form, MoO_4_^−^. In contrast, in acidic soils with a pH below 5.5, Mo availability decreases due to increased adsorption of the anion onto soil oxides (Brinza et al., 2008). Moreover, organic matter in the soil can interact with molybdenum to form complexes, thereby affecting its availability to plants. Soils with high organic matter content can improve Mo retention and enable its slow, sustained release providing a consistent supply for plant uptake. This controlled release can profoundly impact microbial activity and play a crucial role in supporting long-term nutrient cycling within the soil ecosystem. It indicates that Mo has a critical role in proper plant functioning and related biological processes happening in the rhizosphere soil (Rana et al., 2020a). Therefore, understanding the impact of long-term Mo supply on soil microbiota, rhizosphere metabolic profiling, and nutrient acquisition by different crops is highly essential for agriculture sustainability.

The nutrient status in soil can regulate the microbial diversity (Kang et al., 2024). Microbial populations exhibit significant dynamism and self-organization in response to the specific nutrients. As a result, both biotic factors, such as ecological interactions, and abiotic factors, like nutrient availability, shape microbial community composition. Studies on their spatial distribution have shown that distinct microbial arrangements emerge under varying levels of nutrient limitations (Mitri et al., 2016). In nutrient-limited conditions, cooperative behaviors, such as the secretion of metabolites, can maintain diversity (Gandhi et al., 2019; Kang et al., 2024). These shifts in microbial communities reflect the assembly processes that link environmental factors to community structure and function. The most recent development of high-throughput sequencing and culture-independent molecular technologies, using 16S rRNA that encodes the small ribosomal unit of RNA, has provided major insights into bacterial functional population diversity under different fertilizer management practices (Dinsdale et al., 2008; Spor et al., 2011). Microbial diversity analysis not only addresses variations in the function and composition of the bacterial community (Abia et al., 2018) but also describes the association among the external drivers and community components (Langenheder et al., 2010). Molecular tactics may answer the questions about the long-term fertilizer management (Mo-supply) effects on the alteration of soil bacterial diversity and abundance.

Root exudates play significant roles in nutrient acquisition and interaction with rhizosphere microorganisms (Selvakumar et al., 2012). Therefore, it is necessary to analyze root metabolites for an understanding of the interaction between soil microorganisms and roots. Previous studies have designated that plants shape and drive the surrounding microbiome through exudate secretion that specifically represses or stimulates distinctive microbial members of soil. The secretions of root exudates in the rhizosphere depend on the availability of nutrients and the physiological stage of plants (Hartmann et al., 2009; Badri et al., 2013; Chaparro et al., 2013). High nutrient availability positively influences the release of root exudates, whereas low nutrient availability restricts the allocation of plant resources to root exudation and alters the microbiology of the rhizosphere (Liu et al., 2011). For example, during N deficiency, less amino acid is secreted by roots of maize plants. This fluctuation in root exudates could impact the microbial community structure and nutrient uptake by plants (Carvalhais et al., 2013). Recently, “omics” approaches (e.g., LM-MS) are being widely used to describe the different mechanistic alterations happening in the rhizosphere soil for better understating of rhizosphere processes (Urano et al., 2010; Booth et al., 2011). The metabolomic approach can unravel the complex underlying mechanisms in the rhizosphere by profiling root exudates, which play a crucial role in various biochemical processes across biological systems (Patti et al., 2012).

Mo can exist in multiple oxidation states ranging from zero to VI, with VI being the most common in soils. Similar to other metals essential for plant growth, Mo plays a vital role in facilitating redox reactions through specific plant enzymes. However, Mo itself is not directly biologically active. Instead, it is primarily incorporated into an organic pterin complex known as the Mo co-factor. This co-factor is associated with Mo-dependent enzymes, or molybdoenzymes, found across the biological systems of plants, animals, and prokaryotes (Jean et al., 2013). Numerous studies have shown that Mo application enhances nutrient availability in the soil, which can contribute to improved plant performance. These outcomes indicated a synergistic interaction between the application of Mo and other essential elements (Nie et al., 2015). This study highlights the previously underexplored role of Mo in shaping rhizosphere soil biological processes, including variations in metabolites and shifts in bacterial community dynamics, under long-term Mo application. By examining the synergistic effects of Mo on nutrient acquisition, rhizosphere metabolites, and microbial composition in maize and soybean cropping system, the research offers fresh insights into Mo’s contribution to improving soil and crop health aligning seamlessly with the objective of unraveling the interaction between soil chemical and biological indicators.

## 2 Materials and methods

### 2.1 Experimental materials and treatment

A long-term experiment was set up in 2008 at the experimental area of Huazhong Agricultural University, Wuhan, China. Maize and soybean were cultivated in April in an average plot size of 18 m^2^ (9 m × 2 m) and harvested in August and September, respectively. The experiment consisted of two treatments for each crop, i.e., −Mo (Control) and +Mo, and each treatment consisted of three replicated plots. In maize and soybean, both treatments received the NPK@120:80:80 kg/ha as urea, superphosphate, and potassium chloride respectively. Phosphorus (P) and potassium (K) fertilizers were completely applied at the time of sowing, while N fertilizer was applied in two splits, i.e., before sowing and after sowing of crops. The +Mo treatment received the Mo fertilizer as ammonium molybdate (0.41 kg/ha) from June 2009 to October 2013. The available Mo content of soil showed a concentration of 1.80 mg/kg, which crossed the threshold normal demand of plant growth (1 mg/kg in soil) (He et al., 2005). Therefore, we closed the Mo fertilizer supply until the collection of soil samples. The test soil was yellow-brown (Alfisol) from Hubei Province (Xinzhou), China. Basic soil chemical characteristics were as follows: pH 5.64; organic matter, 15.5 g/kg; available N, 80.5 mg/kg; available P, 8.11 mg/kg; available K, 120.6 mg/kg; and available Mo, 0.112 mg/kg.

### 2.2 Sample collection

Plant and soil samples were collected during the maturity stages of crops. Rhizosphere soil samples from maize and soybean fields were collected by gently uprooting four to six plants from each plot, and the collected rhizosphere soils were combined to make a composite sample. The composite sample represents the single sample that was obtained after mixing of representative samples. The composite soil samples were then divided into two sets. One set of composite samples was transferred to ice boxes, where large organic debris was removed. The samples were then thoroughly homogenized, wrapped in aluminum foil, and stored in liquid nitrogen. These samples were transported to the laboratory in liquid nitrogen and stored at −80°C for molecular analysis. The other set of composite samples was air dried at room temperature and manually ground to pass through a 2-mm sieve for chemical analysis of the soil. The harvested plant samples were washed with distilled water and then oven dried (80°C) to achieve constant weight. The dried plant samples were then ground through a stainless-steel grinder for plant chemical analysis.

### 2.3 Soil and plant chemical analysis

The chemical properties of the soil were analyzed by following the methods of Bao (2002). The soil pH was measured in a 1:2.5 water suspension with a pH meter (Mettler Toledo, China) as described by Rana et al. (2018, 2020b). Soil organic matter (SOM) was determined through the dichromate digestion method. Available N was determined by the alkali hydrolysis–diffusion method, and P was determined using the Mo antimony colorimetric method by a spectrophotometer (Lu, 1999); available K was measured using the flame photometric method. The polarographic catalytic wave analysis technique was used to analyze the available Mo content in the soil (Wen et al., 2018). The total N concentrations were determined by following the method of Barbano and Clark (1990). Plant K concentration was determined using a flame photometer (Model 410, USA). P concentration in the digested tissues was determined colorimetrically followed by the Mo blue method (Lu, 1999). The Mo contents in plant samples were determined by polarographic catalytic wave analysis using a JP-2 oscilloscope polarograph according to Ismael et al. (2018); Imran et al. (2019a, 2019b), and Rana et al. (2020c, 2020d).

### 2.4 Bacterial community analysis

Through the PowerMax DNA isolation kit (MoBio Laboratories, Carlsbad, CA, USA), total genomic bacterial DNA was extracted from the soil sample according to the instructions of the manufacturer and stored at −20°C. The quantity and quality of extracted DNAs were measured using a NanoDrop ND-1000 spectrophotometer (Thermo Fisher Scientific, Waltham, MA, USA) and agarose gel electrophoresis, respectively.

The V4 region of bacterial 16S rRNA genes was subjected to PCR amplification through forward primer 515F (GTGCCAGCMGCCGCGGTAA) and reverse primer 806R (GGACTACHVGGGTWTCTAAT) (Caporaso et al., 2011; Ding et al., 2024). The PCR components consisted of 25 μl of PCR Master Mix, 3 μl of each forward and reverse primer (10 μM), 10 μl of DNA template, and 6 μl of ddH_2_O. Thermal cycling comprised a 30-s denaturation at 98°C, followed by 25 cycles consisting of denaturation at 98°C for 15 s, annealing at 58°C for 15 s, and extension at 72°C for 15 s, with a final 1-min extension at 72°C. Agencourt AMPure XP Beads (Beckman Coulter, Indianapolis, IN) were used to purify the PCR amplicons and quantified by the Kit (PicoGreen dsDNA Assay) (Invitrogen, Carlsbad, CA, USA). Amplicons were pooled in equal amounts after the individual quantification step, and then, using the Illumina HiSeq4000 platform, paired-end 2 × 150-bp sequencing was performed at GUHE Info technology Co., Ltd (Hangzhou, China). To process the data, the Quantitative Insights Into Microbial Ecology (QIIME, v1.9.0) pipeline was employed, as described by Caporaso et al. (2010). Briefly, raw sequencing reads that exactly matched the barcode were assigned to each sample and identified as valid sequences. Low-quality sequences were screened by the criteria explained by Gill et al. (2006) and Chen and Jiang (2014): sequences <150-bp length, sequences with an average Phred score of <20, sequences containing ambiguous bases, and sequences containing single nucleotide repeats of >8 bp. Paired-end reads were pulled together using FLASH (Magoč and Salzberg, 2011). OTU picking was performed using Vsearch v1.11.1, which included dereplication, cluster, and detection of chimeras (Rognes et al., 2016). Through default parameters, a representative sequence was selected from each OTU. VSEARCH search was used to perform OTU classification on representative sequences against the Greengenes database. An OTU table was also generated to record the abundance of each OTU in each sample and the classification of these OTUs. All those samples that contained less than 0.001% of the total sequence of OTUs were discarded. To minimize differences in sequencing depth between samples, an average, rounded OTU table was generated by averaging 100 uniformly resampled OTU subsets at 90% of the minimum sequencing depth for further analysis.

### 2.5 Metabolomics analysis

For analysis, 200-mg samples were used, and then 800 μl of methanol and 10 μl of internal standard (2.9 mg/mL, DL-o-chlorophenylalanine) were added. Thereafter, the samples were subjected to vortexing (30 s) and centrifugation (12,000 rpm) at 4°C for 15 min. The supernatant was collected and concentrated by adding 200 μl of methanol and moved to a vial for analysis. The instrument analysis platform was LC-MS (Thermo, Ultimate 3000LC, Orbitrap Elite), while the chromatographic column was C18 [Hypergod C18 (100 × 4.6 mm 3 µm)]. Chromatographic separation conditions were as follows: column temperature: 40°C, flow rate: 0.3 ml/min, mobile phase A: water + 0.1% formic acid, mobile phase B: acetonitrile + 0.1% formic acid, injection volume: 4 ml, automatic injector temperature: 4°C, ESI: heater temperature 300°C, sheath gas flow rate: 45 arb, auxiliary gas flow rate: 15 arb, sweep gas flow rate: 1 arb, spray voltage: 3.0 kV, capillary temperature: 350°C, and S-Lens RF level: 30%. The gradient of the mobile phase is shown in **Supplementary Table S1**.

### 2.6 Statistical analysis

The least significant difference (LSD) test at p < 0.05 was used for mean variances of the data. Statistix 8.1 software (Analytical Software, Tallahassee, FL, USA) was used for statistical analyses of data following analysis of variance. The analyses of sequence data were carried out through QIIME. The OTU table in QIIME was used to calculate the OTU level alpha diversity index. Generated abundance curves of OTU gradients were used to compare the richness and uniformity of OTUs between samples. UniFrac distance metrics were used for beta diversity analysis to study structural changes in microbial communities across samples (Lozupone and Knight, 2005; Lozupone et al., 2007) and visualized by principal coordinate analysis (PCoA) (Ramette, 2007). PCA was also performed based on the compositional profiles at the genus level (Ramette, 2007). The importance of microbiota structure differentiation between groups was evaluated through permutational multivariate analysis of variance (PERMANOVA) (McArdle and Anderson, 2001) using R package (vegan). GraPhlAn (Asnicar et al., 2015) and MEGAN were used to visualize the abundance and taxonomic composition (Huson et al., 2011). To see the unique and shared OTUs between the treatments, a Venn diagram was created using the R package that was constructed based on the presence of OTUs regardless of their relative abundance (Zaura et al., 2009). Based on high-quality sequences, microbial functions were predicted using phylogenetic investigation of communities by reconstruction of unobserved states (PICRUSt) (Langille et al., 2013). The output files were further analyzed using the statistical analysis of metagenomic profiles (STAMP) software package (Parks et al., 2014). Beta diversity and Meta-Storms distance-based functions were created using Parallel-META 3 (Jing et al., 2017). For the metabolite variations, the data were analyzed using feature extraction and pre-processed using SIEVE software, then normalized through Excel 2010 and edited into a two-dimensional data matrix, together with retention time (RT), compound molecular weight (compMW), observed values (sample), and peak intensity. The data were subjected to MVA (multivariate analysis) using the SIMCA-P software (Umetrics AB, Umea, Sweden). OPLS-DA model’s VIP (important variable in projection) value (threshold value >1) and t-test p value (p < 0.05) were used to find differentially expressed metabolites.

## 3 Results

### 3.1 Effect of Mo on soil physiochemical characteristics

The chemical properties of soil changed substantially after long-term Mo applications (**Table 1**). Soil pH showed decreasing and increasing trends for both crops. In particular, there was no significant effect on soil pH. The pH increase was minor under the +Mo treatment in the soybean rhizosphere soil, while in the maize crop, it indicated a reduction difference of 0.12 U under the +Mo treatment. The changes in soil pH were slightly greater in maize rhizosphere compared to those in soybean rhizosphere (**Table 1**). The concentrations of N, P, K, and Mo were significantly (p *<* 0.05) higher in the soil that received treatment with Mo than those in the soil that received treatment without Mo. In addition, P and K concentrations in soybean soils were 15.05% and 9.26% higher in the +Mo treatment compared to those in the −Mo treatment. Conversely, N concentration decreased to 0.66%, whereas in maize rhizosphere soil, we found that Mo supply increased the N, P, and K concentrations up to 5.06%, 10.94%, and 14.52%, respectively.

**Table 1:** Changes in soil NPK, available Mo, and pH under Mo fertilization.

Crop	Treatment	N (mg kg^−1^)	P (mg kg^−1^)	K (mg kg^−1^)	Mo (mg kg^−1^)	pH
Maize	−Mo	50.15 ± 3.43a	80.64 ± 1.47b	238.67 ± 8.98a	0.064 ± 0.009b	4.99 ± 0.087a
	+Mo	52.69 ± 1.03a	89.47 ± 1.26a	273.33 ± 15.62a	0.476 ± 0.039a	4.87 ± 0.013a
Soybean	−Mo	57.00 ± 3.11a	82.68 ± 0.97b	230.33 ± 17.09a	0.068 ± 0.004b	5.22 ± 0.046a
	+Mo	57.38 ± 3.11a	95.13 ± 1.59a	251.6 7± 10.92a	0.519 ± 0.023a	5.28 ± 0.051a

The data are the average of three replicates ± SE from different independent treatments. Different small letters (a, b) in each column are significantly different (*p <* 0.05) among different treatments. −Mo and +Mo indicating the different Mo treatments applied to crops.

### 3.2 Effect of Mo on concentration and accumulation of nutrients in different parts of the plants

**Figure 1:**
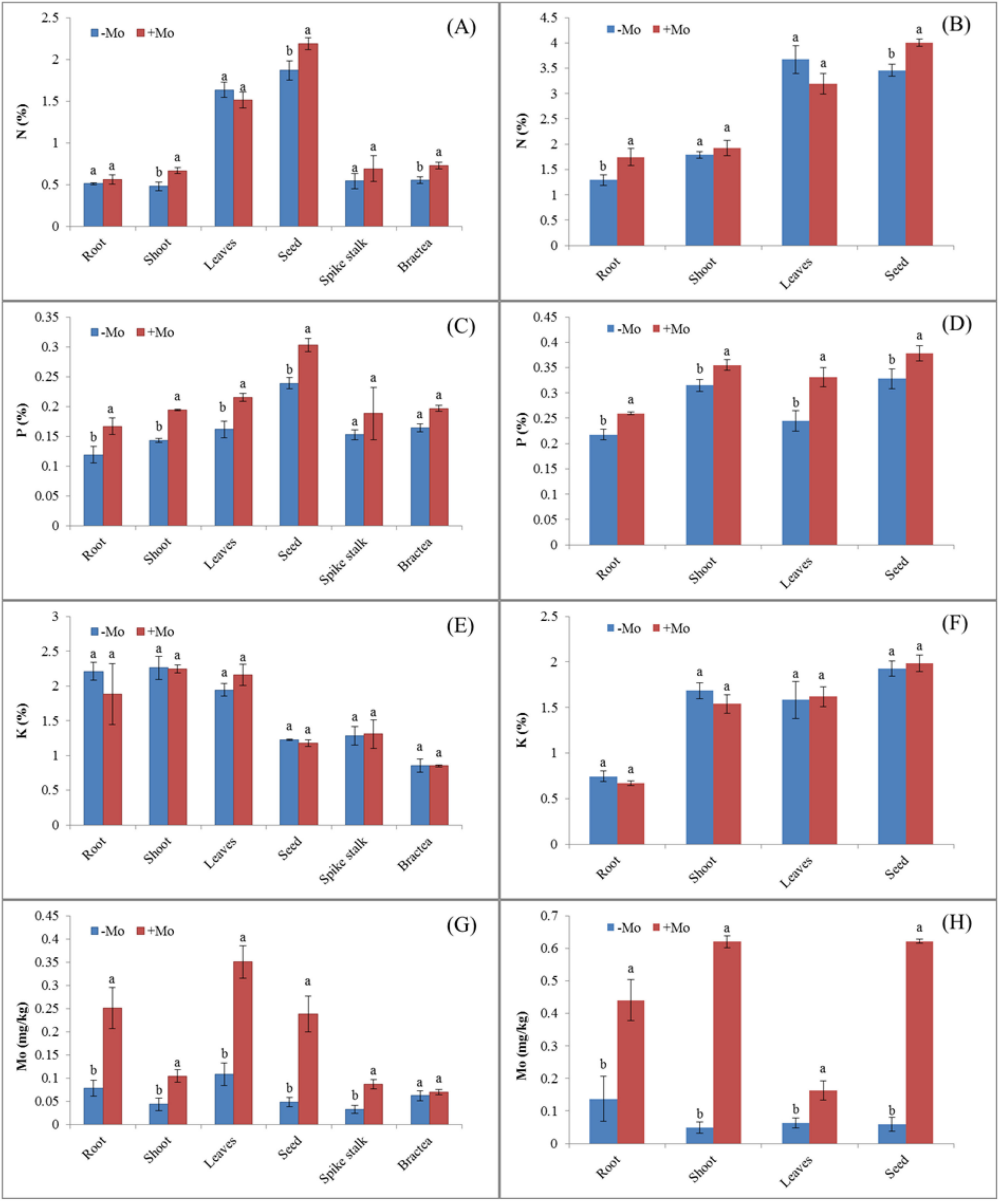
Nutrient acquisition in different parts of maize and soybean crops under Mo application. Treatments: −Mo (without Mo application) and +Mo (with Mo application). Vertical bars represent the standard error of three replicates. Different lowercase letters (a, b) indicate significant differences according to the LSD test (p *<* 0.05). **(A, C, E, G)**, illustrate N, P, K, and Mo acquisition in maize, respectively, while **(B, D, F, H)**, indicate these nutrient acquisitions in soybean crop.

**Figure 2:**
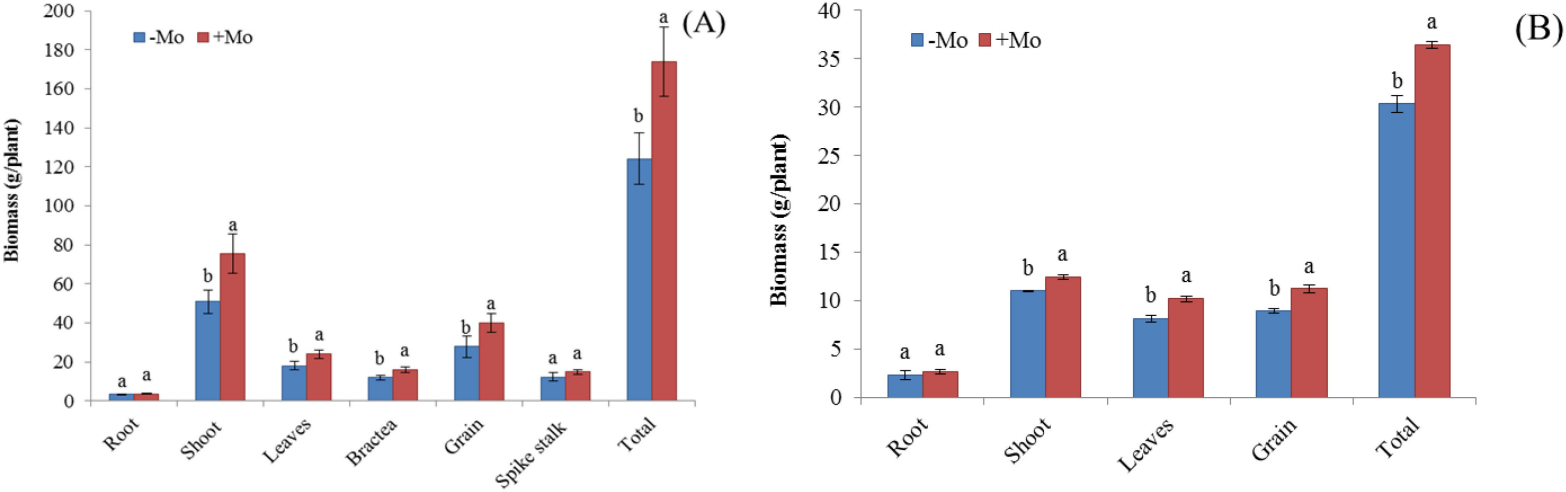
Effect of Mo application on biomass yield components of maize and soybean under +Mo (with Mo application) and −Mo (without Mo application) treatments. Vertical bars represent the standard error of three replicates. Different lowercase letters (a, b) indicate significant differences according to the LSD test (p *<* 0.05). **(A)** shows the treatment effects on maize growth, while **(B)** illustrates the effects on soybean growth.

Maize showed a higher N uptake in all parameters except for the leaves with the +Mo treatment, with significant differences observed in the stem (0.69%), bractea (0.72%), and grain (2.18%) (**Figure 1A**). A similar trend was observed in soybean, with significant N concentration differences in roots and seeds (**Figure 1B**), where N acquisition was higher in soybean than in maize. K concentration decreased in all maize parameters except for the leaves and spikestalk, while it increased in soybean leaves (1.62%) and grains (1.98%) under Mo treatment (**Figures 1E, F**). Mo supply significantly increased P concentration in all plant parts of both crops, except for the maize bractea and spikestalk (**Figure 1C**). Mo concentration also significantly increased in all physical parameters for both crops (**Figures 1G, H**). Nutrient concentrations were generally higher in soybean than in maize. Biomass was significantly higher in maize with Mo application, except for the roots and spikestalk (**Figures 2A, B**). In soybean, Mo supply significantly increased biomass in most plant parts resulting in a higher grain yield compared to those with −Mo treatment.

Nitrogen accumulation in maize crop was significantly higher in stem, seed, and bractea in the +Mo treatment than those in the −Mo treatment. On the other hand, in soybean, a significant difference was observed only in the seeds, with higher nitrogen (N) accumulation in the +Mo treatment. In the other plant parts, N accumulation did not show significant differences between the two treatments (**Supplementary Figure S1**). In maize crops, the stems, leaves, seeds, and bracteas exhibited significantly higher P accumulation in Mo-applied treatment. In soybean, P accumulation was significantly high in all parameters except for the root under the +Mo treatment. No statistically significant difference was observed in K accumulation in the soybean under the Mo fertilizer. Mo accumulation was significantly higher in both crops in all the parameters except for the bractea and seed in maize and soybean, respectively (**Supplementary Figure S1**). Results also indicated that the concentration of nutrients in different parts were higher in the soybean crop than in the maize crop, while the nutrient accumulation results showed an opposite trend. The results indicated that Mo supply played an important role in enhancing nutrient acquisition and improved biomass compared to other treatments.

### 3.3 Response of species richness and bacterial community diversity under Mo supply

**Figure 3:**
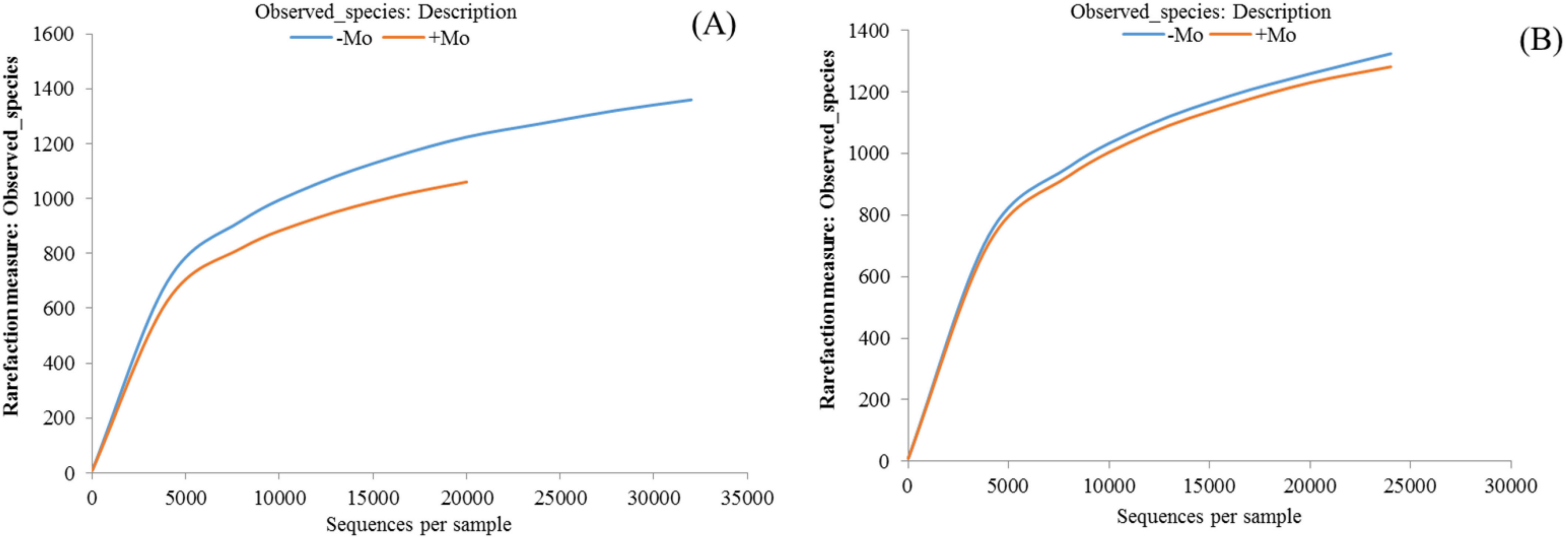
Rarefaction curves showing the observed operational taxonomic units (OTUs) in maize and soybean under +Mo (with Mo application) and −Mo (without Mo application) treatments. **(A, B)** indicate the maize and soybean crops, respectively. The abscissa in the refraction curve represents the number of sequences, while the ordinates indicate the number of OTUs observed. The abscissa position at the end point of the sample curve is the number of sequences of the sample.

The average total valid OTUs were 2,328.66 in maize and 2,350.66 in soybean, with no significant difference observed between crops (ANOVA, p < 0.05). The highest OTU numbers were recorded under −Mo treatment in both crops. Alpha diversity analysis using the Shannon index showed no significant effect of fertilization, though values were higher with +Mo in maize and lower in soybean. Similar trends were observed with the Simpson index (**Table 2**). The Chao 1 estimator also indicated no significant differences between treatments but highlighted higher alpha diversity in soybean rhizosphere soil with Mo application. Rarefaction analysis was carried out with each sample to evaluate whether more sampling would add more OTUs. The rarefaction curve represented that in both crops, −Mo showed a steeper curve, while +Mo indicated a less steep slope (**Figure 3**). Although thousands of tags were observed in both crops, none of the curves appeared to be gentle or reach a plateau indicating high species diversity in the samples. 16S rRNA gene analysis indicated that crop and fertilization can influence the bacterial community taxonomic composition. A Venn diagram shows the degree of interaction of bacterial OTUs among the −Mo and +Mo treatment (**Supplementary Figures S2A, B**). The results clearly demonstrated that 1,080 OUTs were common in maize soil, while 1,086 OUT were similar in soybean crop under both treatments. This indicates that there was much similarity between the crops in the sharing of OTUs among the −Mo and +Mo treatments. In comparison between maize and soybean, it can be seen from the results that soybean exhibited a steeper slope compared to maize (**Figures 3A, B**).

**Table 2:** Effect of the Mo supply on alpha diversity of bacterial community in miaze and soybean soil.

	Treatment	OTU number	Shannon	Simpson	Chao1	OTU sequence
Maize	−Mo	1,341.66 ± 38.32a	7.97 ± 0.076a	0.98 ± 0.001a	1,580.64 ± 15.30a	29,820 ± 4,105.55b
	+Mo	987 ± 177.64a	7.84 ± 0.235a	0.98 ± 0.002a	1,232.44 ± 208.27a	13,803.66 ± 4,873.46a
Soybean	−Mo	1,239.66 ± 65.43a	8.08 ± 0.102a	0.98 ± 0.001a	1,542.85 ± 82.86a	20,677 ± 2,123.98a
	+Mo	1,111 ± 100.58a	7.54 ± 0.273a	0.97 ± 0.006a	1,346.95 ± 82.56a	20,649.66 ± 2,560.76a

The data are the average of three replicates ± SE from different independent treatments. Different small letters (a, b) in each column are significantly different (*p* *<* 0.05) among different treatments. −Mo and +Mo indicate the different Mo treatments applied to crops.

### 3.4 Effect of Mo on bacterial community structure

**Figure 4:**
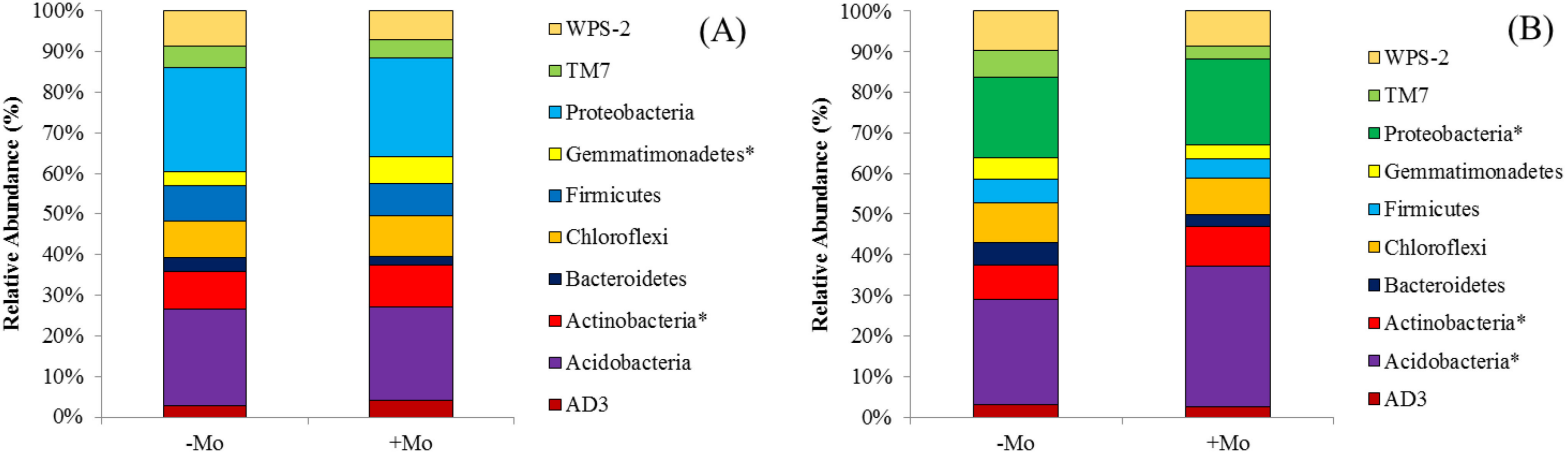
Proportional distribution of bacterial phyla in maize and soybean under +Mo (with Mo application) and −Mo (without Mo application) treatments. **(A, B)** indicate the maize and soybean crops, respectively. The star (*) represents significant differences using the LSD test (p *<* 0.05).

Fertilizer treatments −Mo and +Mo had various effects on the composition of bacteria at the phylum level. The distribution of each phylum was different in each treatment for both crops. The distribution of the top 10 phyla is shown in **Figures 4A, B**. In soybean, *Acidobacteria* was the most abundant (22.30%–35.17%), while in maize, it ranged from 21.30% to 22.57%. In maize, the dominant phylum was *Proteobacteria*, which showed higher values compared to other phyla. In maize and soybean, +Mo showed higher percentages of *Proteobacteria* and *Acidobacteria* compared to the −Mo treatment. These two phyla were found to occupy the subsequent top phyla in all soil samples accounting for 39.43% and 57.74% in soybean with −Mo and +Mo treatments, respectively. In maize, these phyla accounted for 44.51% and 46.64% for both treatments. The Mo supply increased the percentage of *Acidobacteria*, *Actinobacteria*, *Proteobacteria*, and the *Chloroflexi* in soybean (**Figure 4B**). Moreover, in soybean, there was a significant difference among the treatments in the abundance of *Acidobacteria*, *Actinobacteria*, and *Proteobacteria*. In contrast, *Chloroflexi* was more prominent in maize with the Mo application. In maize, *Actinobacteria* and *Gemmatimonadetes* showed significant differences between the two treatments.

At the class level, there was a significant difference between the treatment for *Acidobacteriia*, *Ktedonobacteria*, *iii-8*, *Actinobacteria*, *Thermomicrobia*, *Gemmatimonadetes*, *Deinococci*, *DA052*, and *α*,*β-proteobacteria* in soybean crop. In soybean, four groups of *Acidobacteriia* were found in more abundance with the following order: *Acidobacteriia* > *DA052* > *Acidobacteria-6* > *Solibacteres*. *Acidobacteriia*, *DA052*, and *Solibacteres* was mostly enriched with the +Mo treatment, while the *Acidobacreia-6* was higher in the −Mo treatment of soybean. In miaze, there was a significant difference in *Acidobacteriia*, *Actinobacteria*, *Cytophagia*, *α*, *β*, and *δ-proteobacteria*, *TK-10*, and *4C0d-2* between the −Mo and +Mo treatments. In maize, the increasing abundance order of *Acidobacteria* > *DA052* > *ABS-6* > *Solibacteres* showed a dominant percentage in +Mo treatment. The abundance of the *Proteobacteria* group, including *α*, *β*, *γ*, and *δ-proteobacteria*, was maximum in both crops (**Supplementary Figures S3A, B**). These classes of *Proteobacteria* phyla were higher with the Mo-applied treatments in maize and soybean. *Acidobacteriia* (20.73%), *DA052* (10.67%), and *α-proteobacteria* (11.10%) in soybean and *Acidobacteriia* (12.97%), *α-proteobacteria* (12.90%), *Ktedonobacteria* (7.73%), and *Bacilli* (7.60%) in maize were prominently high with the Mo application (**Supplementary Figures S3A, B**).

**Figure 5:**
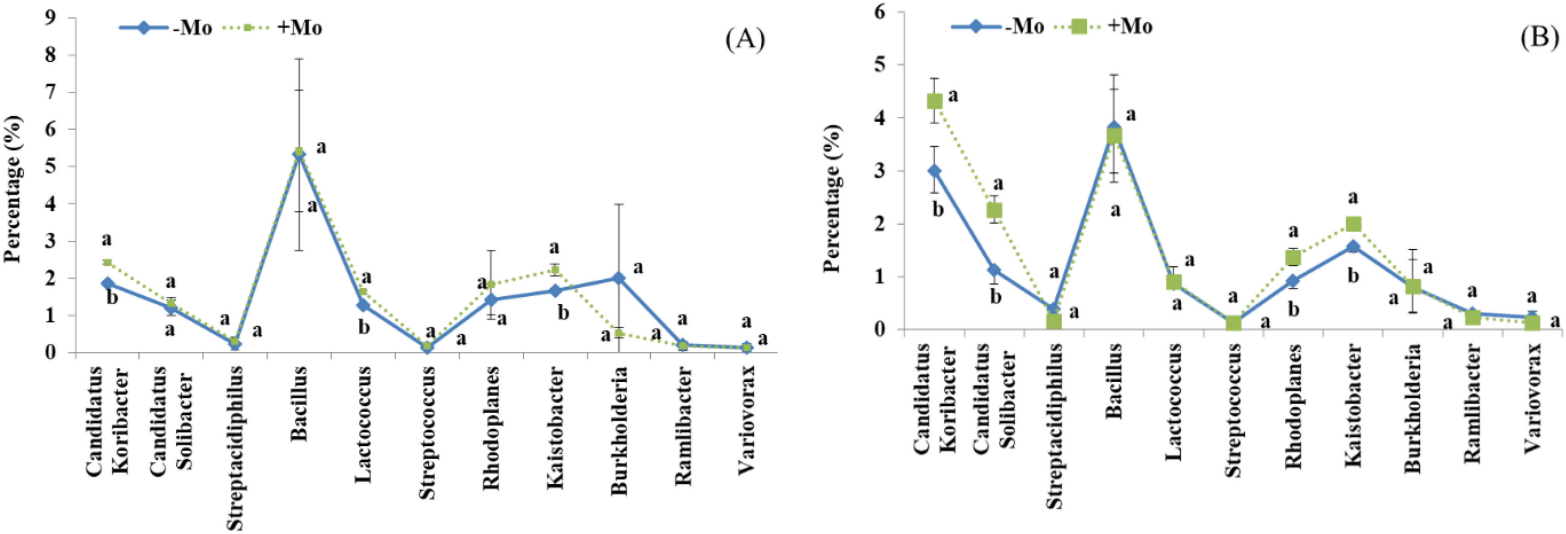
Proportional distribution (%) of the most abundant genera across all samples under +Mo (with Mo application) and −Mo (without Mo application) treatments. Vertical bars represent the standard error of three replicates. Different lowercase letters (a, b) indicate significant differences according to the LSD test (p *<* 0.05). **(A, B)** indicate the maize and soybean crops, respectively.

**Figure 6:**
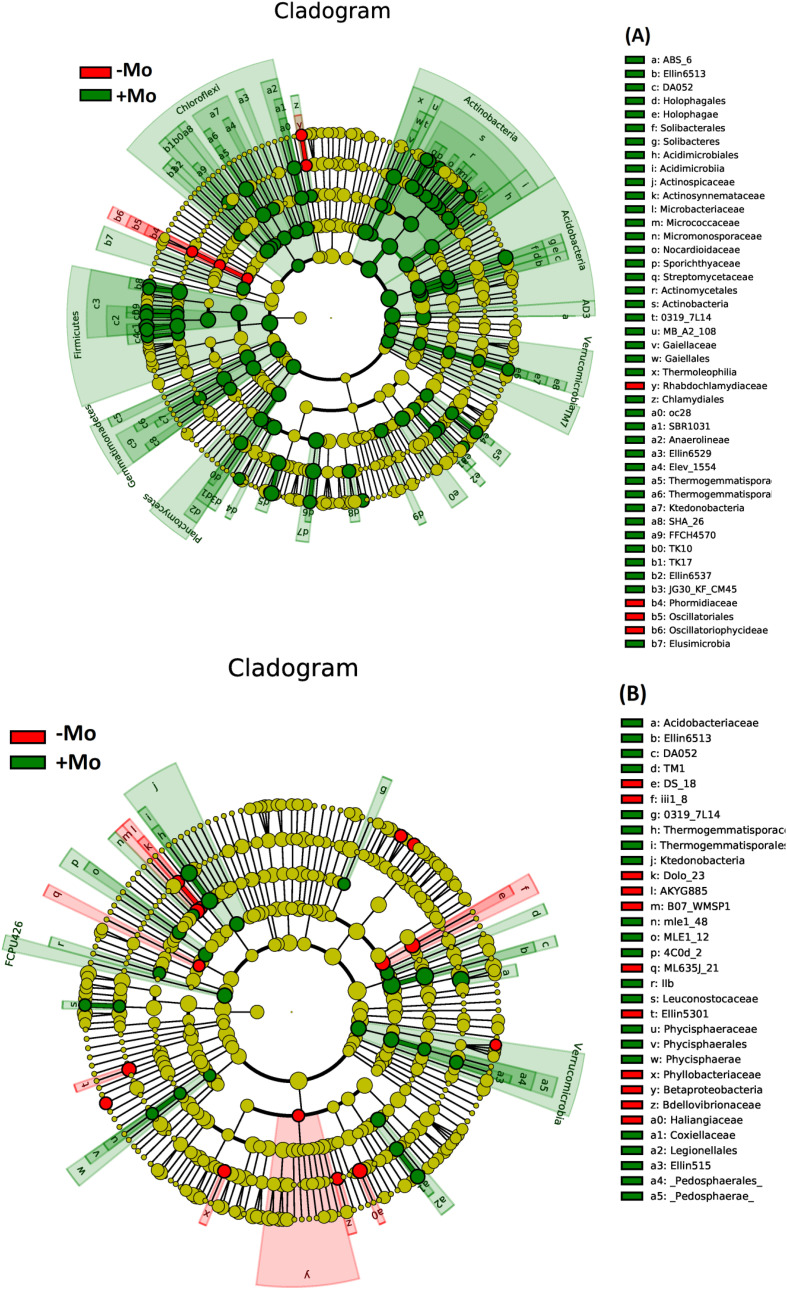
Phylogenetic dendrogram of specific bacteria in maize **(A)** and soybean **(B)** under +Mo (with Mo application) and −Mo (without Mo application) treatments. Different colors represent distinct bacterial groups, while the colored nodes in the dendrogram highlight bacterial groups that play key roles in the corresponding clusters. The species names represented by the letters are detailed in the legend on the right.

**Figure 7:**
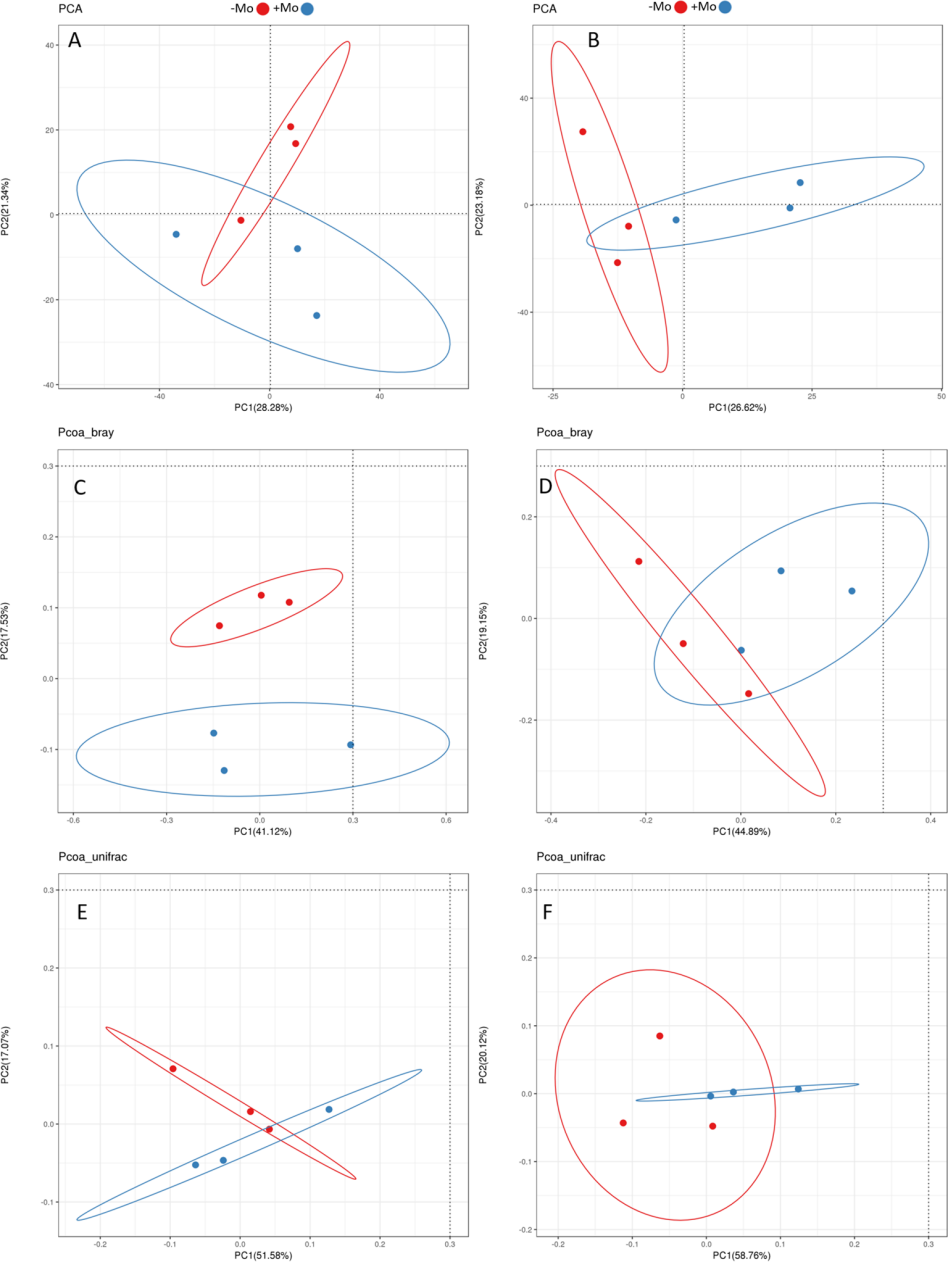
Principal component analysis (PCA) and principal coordinate analysis (PCoA) of beta diversity in soil samples of −Mo and +Mo treatments. **(A, C, E)**, show the variations in maize, while **(B, D, F)**, indicate these changes in soybean crop.

At the genus level, this study showed that the bacterial community composition along with common 11 genera distribution varied between the samples of both crops (**Figures 5A, B**). A significant alteration was observed in the abundance of *Candidatus koribacter*, *Rhodoplanes*, and *Kaistobacter* in the soybean treatment, with these taxa being most abundant in the +Mo treatment. Besides this, *Candidatus koribacter*, *Kaistobacter*, *Lactococcus*, *Rhodoplanes*, and *Burkholderia* were significantly higher under +Mo in maize crop. *Bacillus* and *Candidatus* were the most abundant genera commonly found in all samples (**Figures 5A, B**). The phylogenetic dendrogram of specific bacteria showed significant differences between treatments indicating that Mo supply has an important role in bacterial community composition (**Figures 6A, B**). A plot of the most significant coordinates revealed a clear separation among the treatments (**Figure 7**). There was more variation among the treatments, but when we compared the maize and soybean crops, there was not much higher variation in beta diversity. The community structure of individual samples was compared through principal component and principal coordinate analyses using the weighted Unifrac metric. The PCoA scheme showed that the bacterial community structure in the −Mo treatment was different from that in the +Mo treatment. The first principle (PC1) component could highly decentralize the treatments that were with or without the application of Mo suggesting that the long-term application of Mo altered the bacterial community of rhizosphere soil. Fertilization management showed numerous impacts on bacterial composition at the phylum, genus, and class levels. It is clear form these results that Mo has a significant role in shaping the microbial community structure and diversity not even among the applied treatment but also between the maize and soybean plant soil.

### 3.5 Effect of Mo on metabolite variations

**Figure 8:**
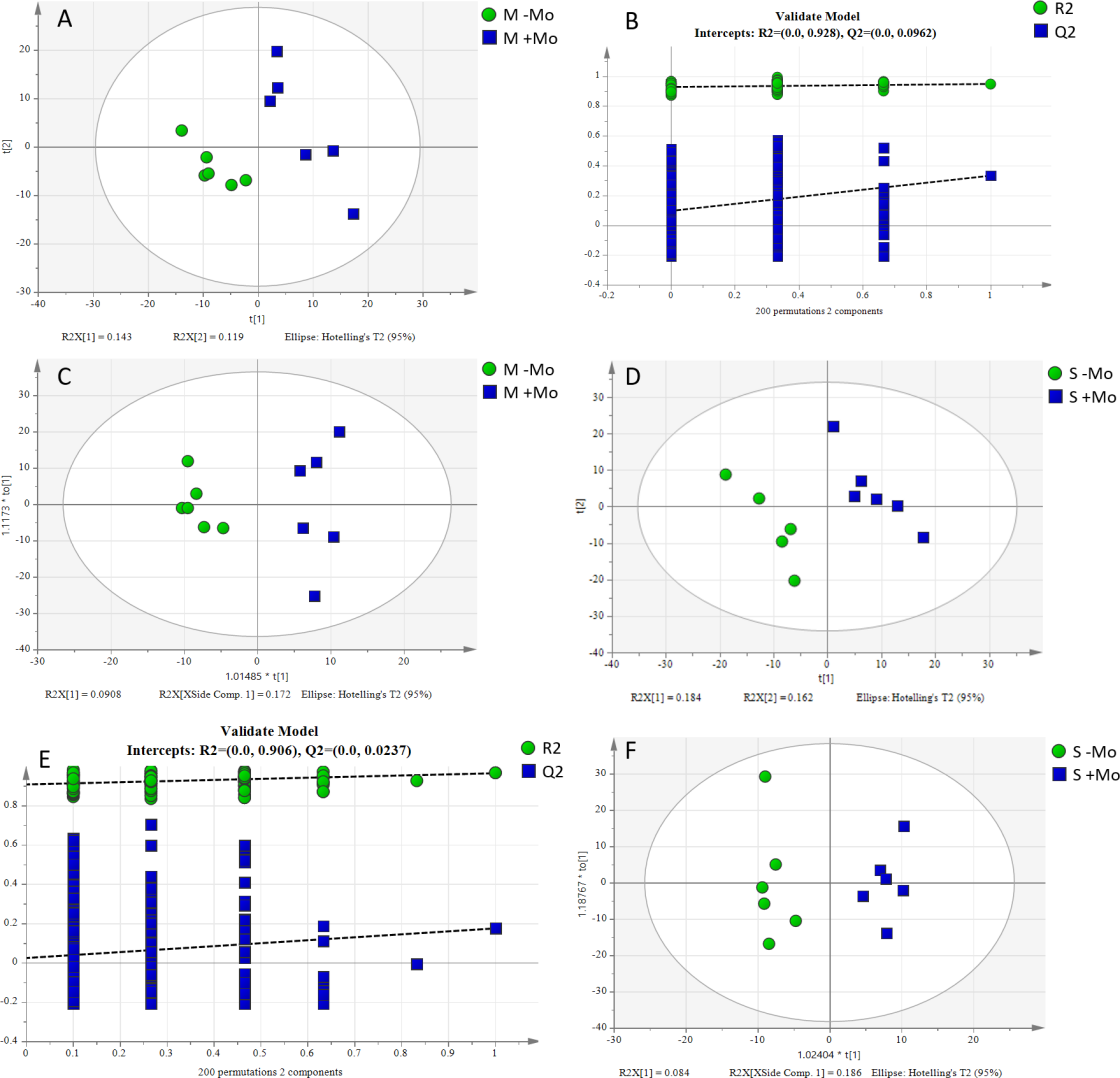
PLS-DA and OPLS-DA score plots and sorting validation diagrams for the −Mo (without Mo application) and +Mo (with Mo application) treatments. **(A, B)** indicate the PLS-DA score chart, while **(C)** represents the OPLS-DA score chart for the maize crop. Similarly, **(D, E)** indicate the PLS-DA score chart, while **(F)** represents the OPLS-DA score chart for the soybean crop.

To assess the impact of Mo application on different metabolites and identify those contributing to significant differences, PLS-DA and supervised OPLS-DA analyses were conducted. The key parameters for judging model quality were R2Y (which indicates the interpretation rate of the model) and the Q2 value (which is the prediction rate of the model). The score chart is shown in **Figure 8** and **Supplementary Figure S4**. The treatments distinctively reacted to a majority of the deviations identified in the treatments across the two axis, and it also can be observed clearly that changes were also prominent under different treatments and crops. Fertilization and crop types resulted in a dramatic shift of metabolites in the rhizosphere. Overall, it could be concluded that fertilizer and crop management have a strong impact on the metabolite variations and their secretions. Different substances mainly included amino acids, organic acids, fatty acids, and so on. The significantly different metabolites in maize and soyabean among the two treatments are shown in **Table 3**. N-acetyl-p-benzoquinonimine, 5-hydroxyindol-2-carboxylic acid, and 4-carboxyphenylglycine were significantly higher in the maize crop under Mo applied treatment compared to those under −Mo treatment (**Table 3**). In soybean, L-serine, phenylacetic acid, PA(20:4(5Z,8Z,11Z,14Z)/22:1(11Z)), oxoglaucine, and Cer(t18:0/24:0(2–OH)) were significantly prominent in the treatment that received the Mo fertilizer compared to those in the −Mo treatment. When we compared the −Mo treatment between the maize and soybean crops, a total of 53 metabolites were found in number that significantly differed in both crops with the same treatment. Seven metabolites were upregulated in the maize crop, while all others were downregulated compared to those in the soybean crop (**Supplementary Table S2**). In the comparison of Mo application in both crops, 70 significantly different metabolites were identified in maize and soybean (**Supplementary Table S3**). Overall, it is concluded that fertilizer management and crops have a strong effect on the metabolites.

**Table 3:** Effect of Mo application on significantly altered metabolites in the rhizosphere soil of maize and soybean under −Mo and +Mo treatments.

	No	Metabolite	VIP	CompMW	RT	Fold M +Mo/M −Mo	T. test
Maize	1	N-Acetyl-p-benzoquinonimine	1.80	149.0472	3.63	1.04	0.047
	2	5-Hydroxyindol-2-carboxylic acid	1.98	177.0421	3.63	1.25	0.025
	3	Choline sulfate	1.99	183.0561	0.87	−0.51	0.024
	4	N-Linoleoyl taurine	1.89	387.2456	3.59	−0.07	0.035
	5	4-Carboxyphenylglycine	2.23	195.0527	3.52	1.41	0.026
	6	18-Hydroxy-9S,10R-dihydroxy-Stearic acid	2.06	332.2556	6.06	−1.48	0.043
	7	3-Oxo-4-pentenoic acid	2.05	114.0318	0.85	−0.93	0.045
	8	L-Erythrulose	2.05	120.0424	0.85	−1.06	0.045
Soybean	9	L-Serine	2.24	105.0422	0.86	0.35	0.009
	10	Phenylacetic acid	2.21	136.0521	4.86	1.44	0.010
	11	γ-Linolenic acid	2.23	278.2239	6.56	−0.67	0.009
	12	MG(18:0/0:0/0:0)	1.83	358.3071	11.33	−0.33	0.048
	13	PA(20:4(5Z,8Z,11Z,14Z)/22:1(11Z))	1.95	778.5541	13.84	0.44	0.032
	14	13S-HOTrE(gamma)	2.84	294.2190	7.51	−1.90	0.004
	15	9-HpOME	2.03	314.2451	6.56	−1.37	0.038
	16	9R,10S,18-trihydroxy-stearic acid	2.28	332.2556	6.06	−1.36	0.034
	17	Oxoglaucine	1.49	351.1097	5.70	1.33	0.013
	18	Cer(t18:0/24:0(2-OH))	1.90	683.6397	13.54	1.04	0.038

VIP, variable importance in projection; CompMW, compound molecular weight; RT, retention time.

## 4 Discussion

This study provides an effective investigation of the Mo influence on crop nutrient acquisition, metabolite variations, and alteration of rhizosphere microbial diversity in maize and soybean. Mo application significantly increased biological yield as well as the concentration and uptake of N, P, and Mo while having no significant effect on K uptake. Similar results were also reported in previous studies (Rawat et al., 2015; Solanki, 2015). Our results revealed that the low mineral nutrient (N, P, K, and Mo) uptake efficiency can be enhanced by Mo application. Notably, Mo application can alleviate the reduced uptake and availability of P in plants and soil by modifying soil chemical properties (**Table 1**). Mo nutrition significantly affects Mo and other nutrient concentrations in soil and plants (Kovács et al., 2015). This may be attributed to the fact that Mo is an essential component of many enzymes and microbes that play a key role in the mobility of mineral nutrients. In fact, our results indicated that Mo concentration in different parts of the plant was significantly correlated with soil Mo availability leading to higher biomass and gain yield. Mo supply significantly increased Mo concentrations in the shoots, leaves, bracteas, spikestalks, and roots in both crops, while the roots showed steadily higher Mo concentrations compared to the shoots. Kádár (1995) and Kovács et al. (2015) also obtained similar results. The steadily higher concentration of Mo in biological parameters indicates that this could be due to the higher N-assimilation, as it is important for the activity and stability of the NR. The interactive effect of Mo on K was significant in soil, which resulted in increased rhizosphere K availability. These results are similar to the findings of Markam et al. (2017).

Phosphorus efficiency increased in soil and plants through the application of Mo. This increase in P facilitated the increased N accumulation and acquisition in the shoots and other parts of the plant. Similar results were also verified in the bean and rice plants by (Høgh‐Jensen et al., 2002). A positive interaction exists between Mo and P due to the formation of anionic complexes and ligand exchange mechanism, which could account for the higher availability of P in rhizosphere soil (Barrow et al., 2005). The increased nutrient availability in the rhizosphere soil could be due to the organic acid secreted by the roots. Overall, it could be concluded that applied Mo increased P contents in maize and soybean crop by enhancing the mineral nutrient availability in rhizosphere soil. This is consistent with other researchers who have found that Mo increases the effectiveness of P in the hydroponic cultivation of rapeseed (Liu et al., 2010).

Synergistic interaction on biological yield among Mo and P fertilizers resulted in the highest biological yield. There was a significant difference in biomass among treatments. In maize, Mo application significantly increased biomass in most of the parameters, except for the roots and spikestalks, where no significant difference was observed. In soybean, Mo significantly increased biomass in all parts except for the roots. Grain yield was higher in the +Mo treatment for both crops compared to that in the −Mo treatment. One possible reason Mo supply enhanced seed yield is the increased Mo and P concentrations in the two crops. This improvement likely boosted compound concentrations, Mo- and P-related enzyme activities (NR, acid phosphatase, glutamine synthetase), photosynthesis, and additional nutrient metabolism ultimately contributing to higher yields (Liu et al., 2009; Yu, 2010; Khan et al., 2014; Manohar, 2014). Mo might also enhance the nitrogenase activity, thereby increasing the supply of N to plants through biological fixation N, thus improving growth and increasing the yield (Biswas et al., 2009). An increase in P availability may be attributed to cell division activity, consequently leading to higher plant dry weight (Tesfaye et al., 2007). These variations may have contributed to higher yield.

Molybdenum played a crucial role in enhancing microbial diversity and nutrient availability. This can be attributed to its function as a co-factor for molybdoenzymes, which are indispensable for key metabolic pathways in both microbes and plants (Mendel and Hänsch, 2002; Sauer and Frebort, 2003; Mendel and Kruse, 2012). Mo improved P availability by reducing its adsorption in acidic soils. This could be due to stimulation of phosphate-solubilizing microorganisms and increasing acid phosphatase activity, thereby releasing P in a form accessible to plants (Brinza et al., 2008; Nie et al., 2015). Furthermore, Mo plays an essential role in enhancing the activity of nitrogen-fixing bacteria and molybdoenzymes, such as nitrogenase and nitrate reductase, both of which are critical for nitrogen cycling and microbial metabolism (Kaiser et al., 2005). These multiple actions of Mo might explain the observed enhancements in nutrient availability, accumulation, and microbial diversity in this study. The Mo-induced secretion of organic acids and metabolites created a favorable environment for microbial growth further boosting nutrient availability and improving soil health. Collectively, these effects of Mo on microbial processes, enzymatic activities, and soil properties promote efficient nutrient cycling and enhance nutrient acquisition by plants.

We performed microbial diversity analysis to determine the composition and diversity of bacterial community responses. This method has been used to evaluate the fertilization effect on plant and rhizosphere soil (Zhao et al., 2014). As shown by the number of OTUs, our long-term fertilization experiments did not significantly affect the richness and diversity of the bacteria. Previous studies have shown that the effects of fertilization on soil bacterial diversity are inconsistent (Coolon et al., 2013). Overall, significant variations in bacterial community composition were observed at the phylum, genus, and class levels. Although thousands of tags were identified per sample, no rarefaction curve reached a plateau indicating that no reasonable bacterial community was sequenced (Sengupta and Dick, 2015; Daquiado et al., 2016). Several studies (Youssef and Elshahed, 2008; Morales et al., 2009) have revealed that the analyzed sequences number per sample affects the number of OTU predictions. Generally, the fewer sample sequences from the Mo treatment led toward a smaller curve progression and a fewer number of predicted OTUs. The variances in soil bacterial diversity might largely be explained by differences in effective nutrient concentrations in soil. Earlier studies have presented that nutrient availability (Eo and Park, 2016) and crop types are significant regulators of soil microbial community activity and composition (Suleiman et al., 2013).

The distinctive configuration of the bacterial community in −Mo and +Mo treatment in both crops inferred that fertilization deficiency in one of the constituents may alter the bacterial community composition potentially leading to differences in nutrient availability, uptake, and crop use efficiency. Soil microorganisms could facilitate nutrient mineralization. These aspects of microbiota could account for increased mineral nutrition and biomass yield in our experiment due to Mo supply. *Proteobacteria* and *Acidobacteria* were the dominant phyla in all the treatments in both maize and soybean especially in Mo-treated treatments. These results are in line with the findings of previous studies (Ahn et al., 2012), as they found a high abundance of these phyla with other nutrients. The higher abundance of *Proteobacteria* could be attributable to the fact that most of the bacterial groups that are functionally diverse belong to the leading phyla of *Proteobacteria* and *Gram-negative* bacteria (Gupta, 2000). Specifically, in the treatments receiving Mo fertilizer, the four *Proteobacteria* groups (*α-proteobacteria*, *β-proteobacteria*, *γ-proteobacteria*, and *δ-proteobacteria*) were the most abundant. Remarkably *Actinobacteria* are thought to actively participate in organic matter degradation (Ahn et al., 2012), and these were prominently high in treatments receiving Mo. Thus, the higher organic matter may play a role in the increased mineral nutrient availability. Metabolites produced by certain phyla may help in maintaining other bacterial populations enhancing nutrient acquisition and crop growth (Sun et al., 2014). Mo supply can improve soil chemical properties supporting beneficial microbial activity in the rhizosphere. *Acidobacteria*, known for thriving in resource-poor environments (Fierer et al., 2007), showed a slight increase with Mo application in our study. This phylum, difficult to cultivate and poorly understood (Ward et al., 2009), may respond variably to environmental changes (Fierer et al., 2007). Both *Acidobacteria* and *Actinobacteria*, along with *Proteobacteria*, increased significantly with Mo application, while they were lower in the −Mo treatments of soybean rhizosphere soil. In case of maize, *Actinobacteria* and *Gemmatimonadetes* were significantly increased with the Mo application than with the −Mo treatment. These phyla in soil environments have been consistently identified as the key divisions of bacteria (Janssen, 2006). The relative abundance response pattern of *Firmicutes* observed in this study was non-linear; such a response has not been previously observed. In +Mo treatment, the *Firmicutes* relative abundance was lower in soybean, while it increased in maize with the same treatment. It could be concluded that the crop type have an effect on relative abundance. Schreiter et al. (2014) described that this phylum in the rhizosphere is more abundant than in non-rhizosphere soil suggesting the constructive influence of plants. Interestingly, the percentage of *TM7* was increased only with −Mo treatment, while it was lower with Mo supply in our study. This proposes that different groups of bacteria might play a parallel role in this treatment regarding the lower availability of nutrients in rhizosphere soil through negative effects. The candidate division *TM7* and family *Sphingomonadaceae* (Phylum *Proteobacteria*) are known to be involved in toluene and benzene degradation (Xie et al., 2011). In terrestrial ecosystems, fluctuating nutrient supply and lower pH are determinants of microbial communities (Zhang et al., 2013). Bacteria belonging to *Chloroflexi* are the main degradation agents of polysaccharides (Schreiter et al., 2014). Fierer et al. (2012) described that after adding N, the proportion of bacteria belonging to these phyla decreased. However, in our results, it is shown that it increases with the application of Mo.

At a lower taxonomic level, the diverse responses of the genus reveal the specific adaptation of bacterial communities to fertilization. *Candidatus Koribacter*, *Rhodoplan*, and *Kaistobacter* were significantly higher in both crops under Mo-applied conditions. The higher abundance of these groups may have contributed to the distinctive biological activity of the communities compared to that in the −Mo treatment. Results suggested that the abundance and relative proportion of these similar taxa having the same function could be used as markers for nutrient availability and soil fertility (Soman et al., 2017). This shift in microbial community structure may have contributed to increased mineral nutrient availability in the rhizosphere soil, as well as higher grain yield and biomass in maize and soybean crops under the +Mo treatment. It has been reported that *Actinobacteria*, *Alphaproteobacteria*, and *Betaproteobacteria* follow copiotrophic lifestyles (Acosta-Martínez et al., 2010; Singh et al., 2010). The copiotrophic environment could be considered in the Mo applied system, which has resulted in higher nutrient cycling due to the activity of phosphatase and phosphodiesterase. The richness of these microbial groups can improve essential nutrient cycling, which may result in increased crop productivity and soil fertility (Lesaulnier et al., 2008; Daquiado et al., 2016). Our findings suggest that Mo application plays a pivotal role in shaping microbial community composition and abundance ultimately contributing to enhanced nutrient use efficiency and improved crop growth and development. Specifically, the shift from a −Mo to a +Mo system appears to alter bacterial community abundance, which is likely due to changes in nutrient availability in the rhizosphere of maize and soybean crops. These results also answer the questions about the mechanisms by which Mo application influences specific bacterial phyla, genera, and classes. One possible explanation is that Mo alters key soil chemical properties, such as O.M content, pH, and available P, creating a favorable environment for certain microbial groups. Another potential mechanism is the suppression of competing bacterial populations through biological activities, such as the production of inhibitory substances or competition for limited resources. Further studies are needed to disentangle the relative contributions of abiotic factors and biotic interactions in driving these changes.

It has been observed that Mo supply may influence the composition and quantity of root exudates impacting the nutritional status of plants and availability of mineral nutrients in the rhizosphere soil. This, in turn, led to alterations in the bacterial community composition in maize and soybean crops. Mo application enhanced the secretion of different metabolites in rhizosphere soil, which impacted the microbiome and promoted the transformation of insoluble nutrients into soluble form. Similar results are also reported in a previous study (Qin et al., 2023). It could be possible that variation in the nutrients resulting from Mo application may have altered the metabolites in the rhizosphere soil. Therefore, the alteration in metabolites may have led to changes in the bacterial community composition. The researcher has reported that Mo supply could enhance the availability of P in the soil through increased secretion of malic acid and succinic acid in different crops. In plants, the lack of L-serine-derived molecules can have serious consequences. For example, a deficiency in phosphatidylserine, a relatively small plant cell lipid, leads to altered microspore development. The mutant deficient in serine palmitoyl transferase, which condenses L-serine and palmitoyl-CoA, participates in the first step of sphingolipid biosynthesis showing embryonic and male gametophyte lethality. Due to a decrease in sphingolipid content, the homeostasis of plant mineral ions changes steadily, and the plant cannot survive (Chao et al., 2011). L-serine is also critical for the transfer regulation of the methyl group by giving tetrahydrofolate metabolism along with C1 units. Serine is also involved in phospholipid formation required for cell production. Phenylacetic acid has been found to be an active auxin (a type of plant hormone) (Wightman and Lighty, 1982). Amino acids secreted by the roots were high in the Mo treatment. 4-Carboxyphenylglycine, PA(20:4(5Z,8Z,11Z,14Z)/22:1(11Z)), oxoglaucine, and Cer(t18:0/24:0(2-OH)) were upregulated significantly in the Mo-applied treatment in maize and soybean. These metabolites mainly consisted of organic acids, amino acids, and lipids. Generally, plants compositionally yield a varied array of diverse low-molecular natural products (>100,000) identified as secondary metabolites (Bais et al., 2006). Early studies showed that gluconic acid, glucuronic acid, and aldehyde sugar acids can be complexed with Mo (Sawyer, 1964; Ramos et al., 1997). Organic acids chelate Mo in soil to reduce the leaching loss of Mo in soil (Wichard et al., 2009). Many of these metabolites in +Mo treatment have a carboxyl group structure suggesting that some of the compounds might be involved in the transport of molybdate. This mechanism could contribute to the higher biomass and grain yield observed in maize and soybean crops. These secondary metabolites and their functions in the rhizosphere of maize and soybean are not well understood. Organic substances released from the roots can also accelerate O.M degradation and stimulate the rhizosphere microorganisms to dissolve insoluble minerals. Root exudates can stimulate the transformation of O.M leading to improved availability of P (Chen et al., 2014). The stimulation of microbial activity in the rhizosphere could activate the nutrient cycling or can speed up the rhizosphere priming effect.

The findings of this research have significant implications for sustainable agriculture, particularly for maize and soybean cultivation in acidic soils. Molybdenum application emerges as a promising, eco-friendly strategy to enhance crop productivity by improving nutrient availability and uptake, especially P, a nutrient often limited in such soils. Mo plays an important role in N metabolism, particularly through its involvement in key enzymes (NR and nitrogenase), which can enhance N use efficiency. This is evident from the increased N availability and uptake observed in our study benefiting both leguminous (Soybean) and non-leguminous crops (Maize). Additionally, Mo application promoted a healthier rhizosphere by improving the microbial diversity and activity aligning with sustainable farming objectives by improving soil fertility, nutrient availability, and their uptake. Incorporating Mo into nutrient management strategies will offer a practical approach to achieve higher yields, preserving soil health, and contributing to global food security through environmentally sustainable practices.

This research provides valuable insights into how Mo influences crop yields, soil microbial diversity, and metabolite variations; however, it is important to recognize its limitations. First, the study was conducted in a specific region with acidic soil, which may restrict the broader applicability of the findings to other soil types or climatic conditions. Expanding future research to include a variety of soil types and environmental settings would enhance the generalizability of the results. Additionally, the 16S rRNA method was employed to analyze microbial communities. While this method is effective for taxonomic profiling, it does not capture the functional complexities of microbial processes affected by Mo. Employing advanced techniques, such as metagenomics or meta-transcriptomics, in future studies could provide deeper insights into the functional dynamics within microbial communities. Furthermore, although metabolite profiling revealed significant changes in response to Mo, the specific roles of these metabolites in plant–microbe interactions and nutrient uptake pathways remain unexplored. Further biochemical and molecular investigations are required to elucidate these mechanisms. Despite these limitations, the findings emphasize the critical role of Mo in improving soil nutrient availability and crop performance. They also highlight the need for continued research to address these gaps and expand our understanding of Mo’s contributions to agricultural ecosystems.

## 5 Conclusion

This study demonstrates the critical role of Mo in improving microbial diversity, nutrient availability, and nutrient uptake in the rhizosphere soils of maize and soybean. Mo application significantly increased biological yield and enhanced the concentrations and uptake of N, P, and Mo, while K uptake remained unaffected. The findings revealed that Mo amendments altered microbial community structures, particularly increasing the abundance of dominant phyla such as *Proteobacteria* and *Acidobacteria*. These changes were associated with enhanced metabolite secretion in the rhizosphere promoting microbial activity and nutrient availability. This led to improved nutrient acquisition and a substantial increase in grain yield. These results underscore the importance of Mo in optimizing soil–plant–microbe interactions and highlight its potential for improving crop performance in acidic soils. Future research should explore the effects of Mo on soil geochemical health, microbial functionality, and its application across diverse soil types and cropping systems.

## Data Availability

The original contributions presented in the study are included in the article/**Supplementary Material**. Further inquiries can be directed to the corresponding author.
