# Supplementary material for: Effect of molybdenum supply on crop performance through rhizosphere soil microbial diversity and metabolite variation
Source: Front Plant Sci. 2025 Jan 28;15:1519540. doi: 10.3389/fpls.2024.1519540 (PMC11811785; doi:10.3389/fpls.2024.1519540)
Supplement: Supplementary file 1 [file DataSheet1.docx]

**Effect of Molybdenum Supply on Crop Performance through Rhizosphere Soil Microbial Diversity and Metabolite Variation**

Muhammad Shoaib Rana^1,2^, Dikhnah Alshehri^3,4^, Rui-Long Wang^1^, Muhammad Imran^5^, Yousif Abdelrahman Yousif Adellah^6^, Faiz Ur Rahman^7^, Marfat Alatawy^3^, Hanaa Ghabban^3,4^, Amany H. A. Abeed^8^, Cheng-xiao Hu^2^*

**Supplementary Data:**


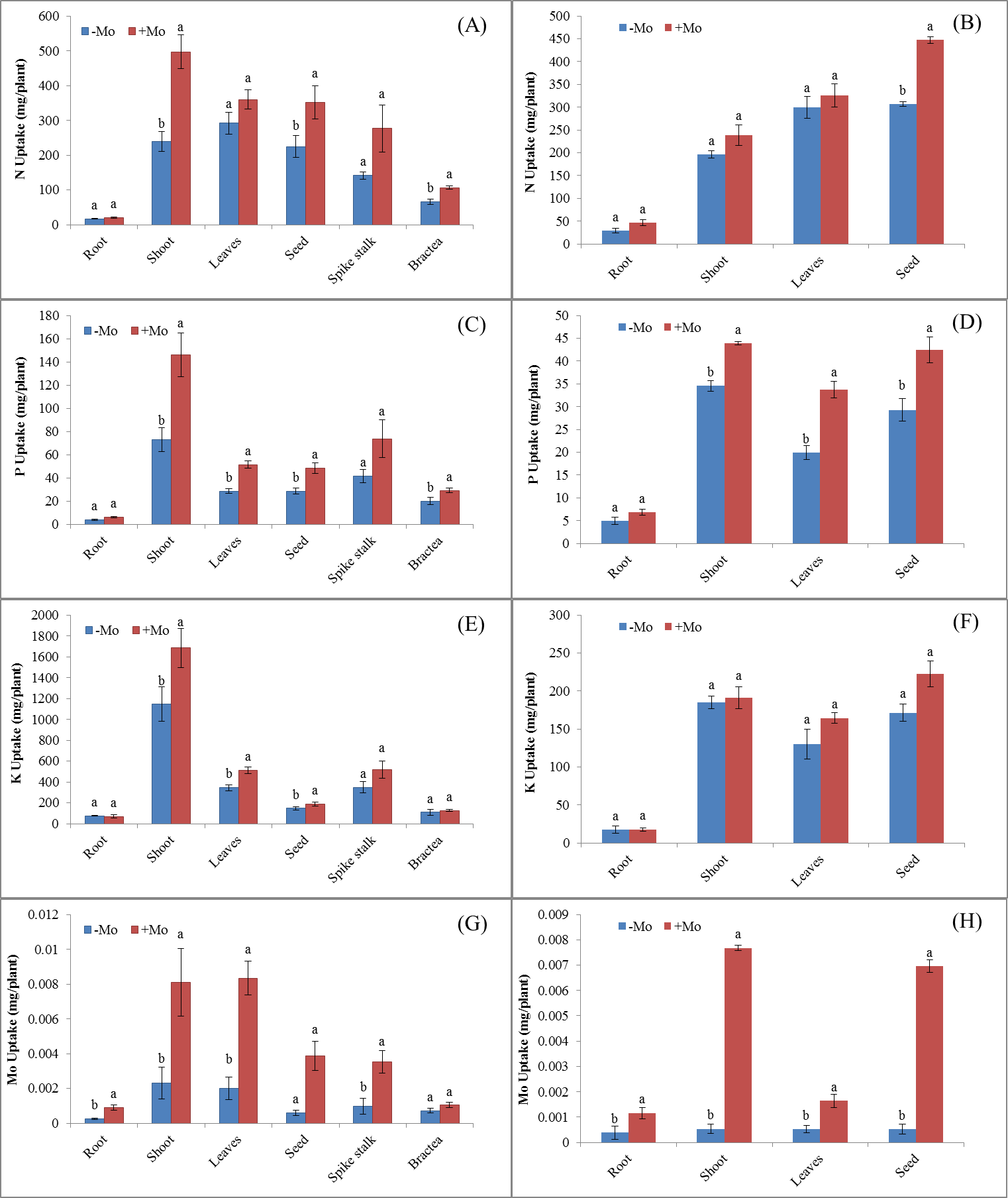


**Fig. S1** Impact of molybdenum (Mo) application on nutrient accumulation in different plant parts, comparing Mo (+Mo) and no Mo (-Mo) treatments. Vertical bars represent the standard error of the mean from three replicates. Different lowercase letters (a, b) above the bars indicate significant differences based on the LSD test (*P <* 0.05). Fig A, C, E, G indicate the accumulation of NPK and molybdenum in maize while B, D, F, H figs. showing the accumulation in soybean crop.


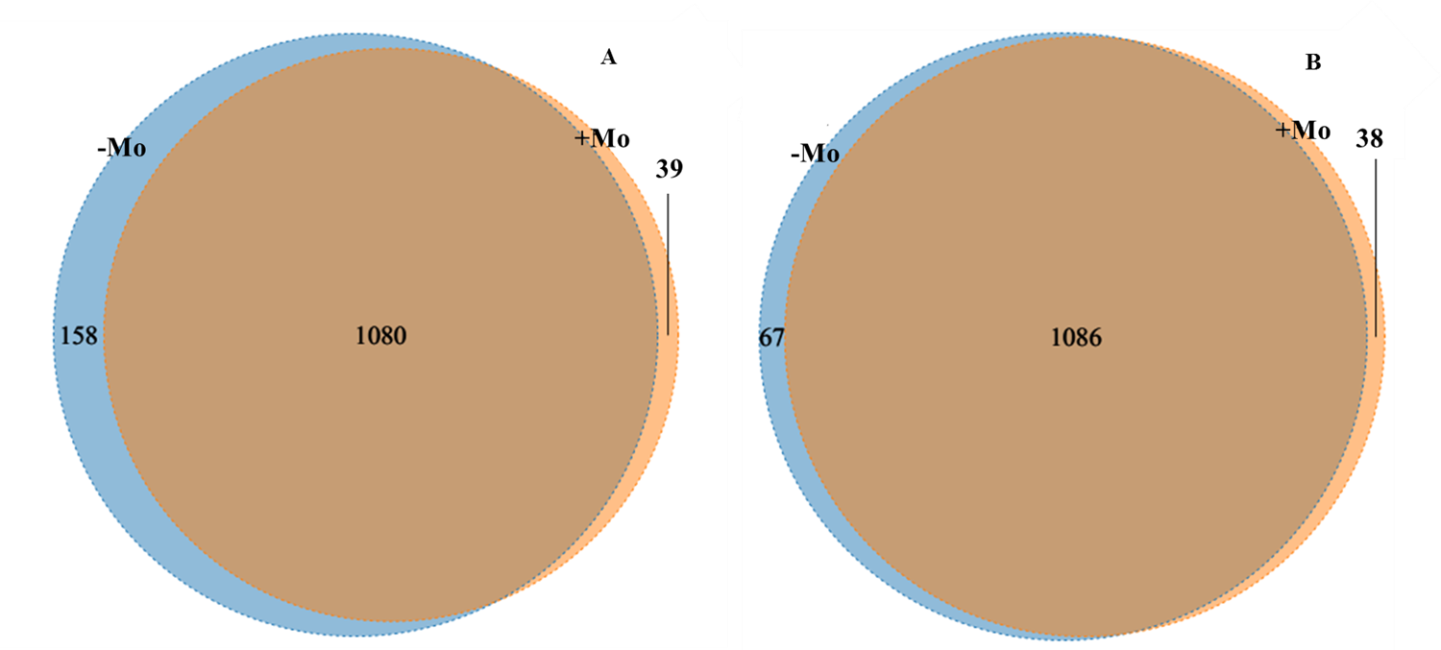


**Fig. S2** Venn diagram displaying the degree of interaction of bacterial OTUs between -Mo and +Mo treatments. Figure A shows the interactions in maize, while figure B represents the interactions in soybean. The overlapping regions indicate the number of shared OTUs, while the non-overlapping regions represent the unique OTUs specific to each treatment.


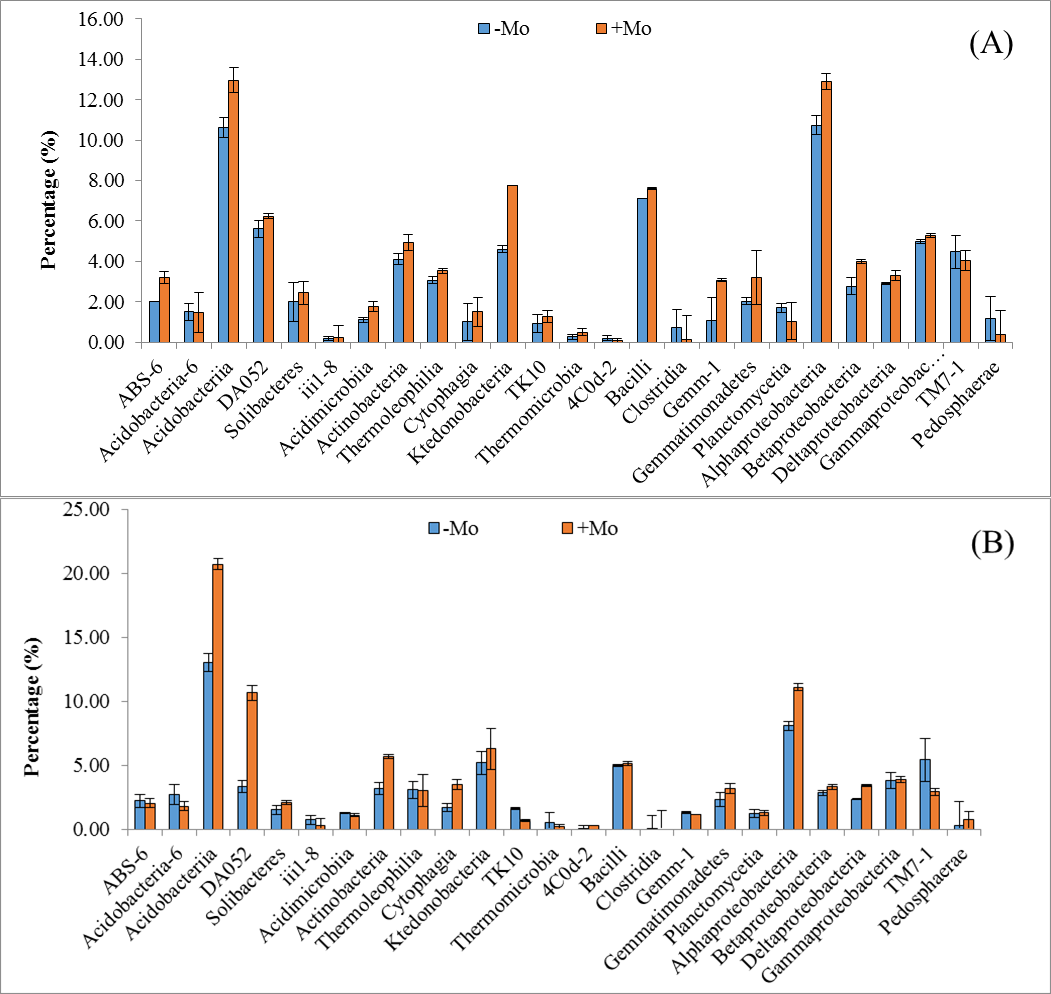


**Fig. S3** Impact of Mo application on bacterial class-level composition. The -Mo and +Mo treatments refer to conditions with and without Mo application, respectively. Figure A and B show the comparison of the percentage of bacterial classes under -Mo and +Mo treatments in maize and soybean, respectively. Vertical bars above the mean indicate the standard error of the mean from three replicates.


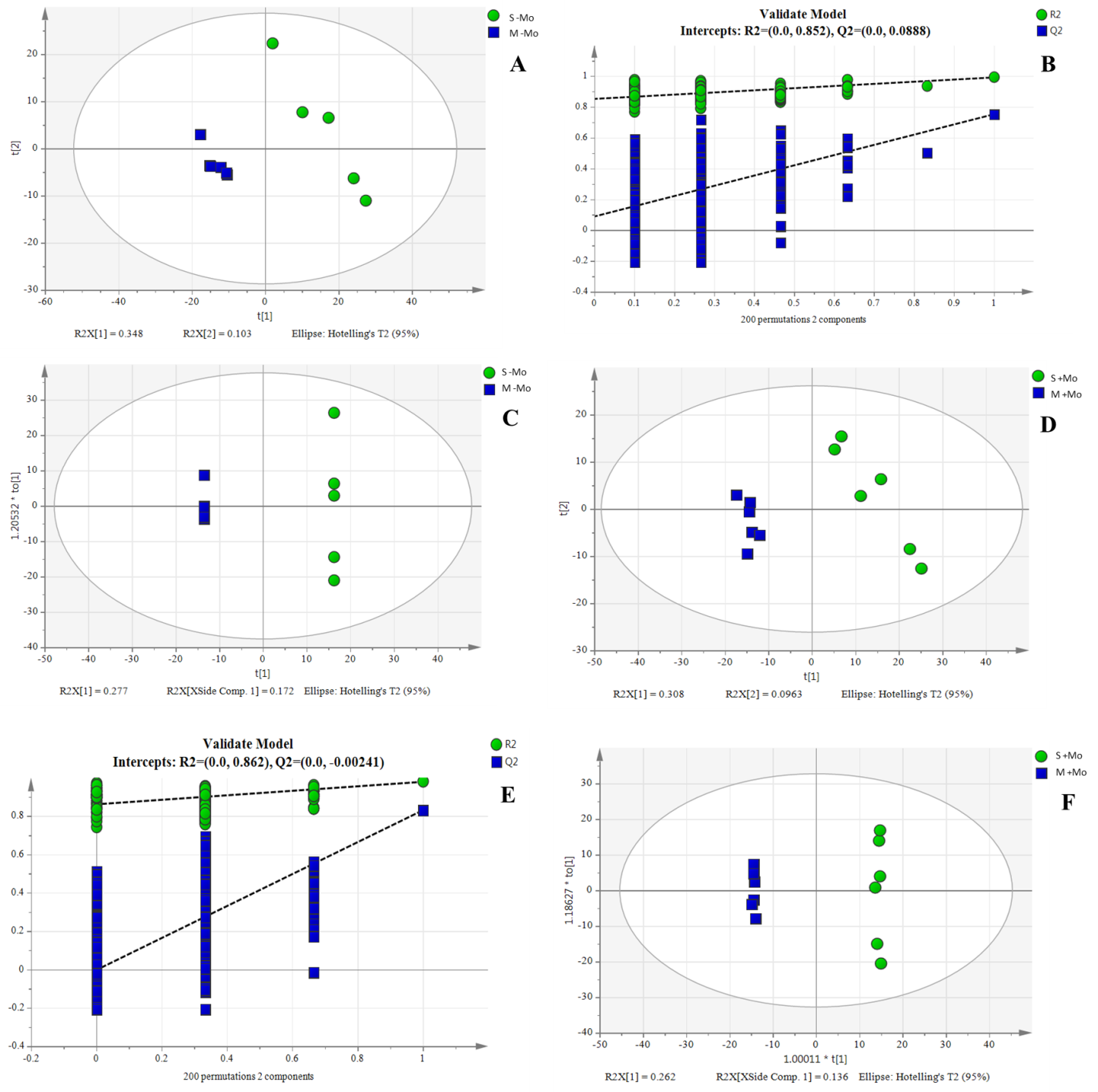


**Fig. S4**  PLS-DA and OPLS-DA score charts and sorting validation diagrams comparing -Mo and +Mo treatments in maize and soybean crops. In the maize crop, M-Mo and M+Mo represent treatments without and with molybdenum application, respectively. Similarly, in the soybean crop, S-Mo and S+Mo represent treatments without and with molybdenum application. Fig AB are indicating the PLS-DA score chart while C represents the OPLS-DA score chart for the maize crop. Similarly, Fig DE are indicating the PLS-DA score chart while F are representing the OPLS-DA score chart for the soybean

**Table S1** The gradient of mobile phase

| Time (min) | Flow rate (mL/min) | A (%) | B (%) |
| --- | --- | --- | --- |
| 0 | 0.3 | 95 | 5 |
| 2 | 0.3 | 95 | 5 |
| 12 | 0.3 | 5 | 95 |
| 15 | 0.3 | 5 | 95 |
| 17 | 0.3 | 95 | 5 |

Table S2 Effect of -Mo treatment on significantly altered metabolites in the rhizosphere soil of maize and soybean crops

| No. | Metabolite | VIP | CompMW | RT | Fold M -Mo/S -Mo | T.test |
| --- | --- | --- | --- | --- | --- | --- |
| 1 | Sucrose | 1.80 | 342.1151 | 0.87 | -0.45 | 0.002 |
| 2 | 1α-hydroxy-25-methoxyvitamin D3 | 1.50 | 430.3432 | 14.52 | -1.03 | 0.022 |
| 3 | Indole | 1.75 | 117.0575 | 3.40 | -0.56 | 0.004 |
| 4 | L-Valine | 1.58 | 117.0785 | 17.12 | -0.14 | 0.014 |
| 5 | N-Acetyl-p-benzoquinonimine | 1.81 | 149.0473 | 3.84 | 2.94 | 0.002 |
| 6 | Salicyluric acid | 1.78 | 195.0526 | 3.63 | 3.55 | 0.003 |
| 7 | 5-L-Glutamyl-taurine | 1.74 | 254.0561 | 4.28 | -6.70 | 0.004 |
| 8 | Adenosine | 1.91 | 267.0963 | 1.21 | -1.49 | 0.001 |
| 9 | Homolanthionine | 1.52 | 268.0729 | 5.12 | -6.34 | 0.020 |
| 10 | 4,8 dimethylnonanoyl carnitine | 1.75 | 329.2556 | 6.11 | -0.18 | 0.004 |
| 11 | 9-deoxy-9-methylene-PGE2 | 1.56 | 350.2446 | 7.12 | -1.57 | 0.015 |
| 12 | MG(0:0/20:4(5Z,8Z,11Z,14Z)/0:0) | 1.45 | 378.2760 | 9.17 | -1.65 | 0.028 |
| 13 | PA(14:1(9Z)/0:0) | 1.90 | 380.1955 | 6.53 | -2.20 | 0.001 |
| 14 | PA(O-16:0/0:0) | 1.41 | 396.2650 | 4.82 | 1.26 | 0.036 |
| 15 | Vitamin D2 | 1.36 | 396.3379 | 15.16 | -0.63 | 0.044 |
| 16 | 1-Hydroxyvitamin D5 | 1.65 | 428.3638 | 12.98 | -0.75 | 0.009 |
| 17 | 26,27-Dihomo-1α-hydroxyvitamin D2 | 1.36 | 440.3638 | 8.97 | -5.11 | 0.044 |
| 18 | (22E,24E)-1α,25-dihydroxy-26,27-dimethyl-22,23,24,24a-tetradehydro-24a-homovitamin D3 | 1.57 | 454.3434 | 8.76 | -1.57 | 0.015 |
| 19 | PA(20:0/0:0) | 1.56 | 466.3067 | 10.14 | -0.93 | 0.016 |
| 20 | Cer(d14:2(4E,6E)/20:1(11Z)) | 1.87 | 533.4781 | 15.13 | -1.36 | 0.001 |
| 21 | Chloramphenicol palmitate | 1.84 | 560.2406 | 10.52 | -1.01 | 0.002 |
| 22 | Cer(t18:0/18:0(2OH)) | 1.35 | 599.5469 | 17.03 | -1.27 | 0.045 |
| 23 | PA(12:0/18:3(6Z,9Z,12Z)) | 1.34 | 614.3947 | 12.54 | -3.15 | 0.049 |
| 24 | PA(12:0/19:0) | 1.49 | 634.4548 | 15.30 | -1.26 | 0.023 |
| 25 | PA(16:0/16:0) | 1.33 | 648.4706 | 15.50 | -0.96 | 0.050 |
| 26 | PG(12:0/18:4(6Z,9Z,12Z,15Z)) | 1.38 | 686.4137 | 11.54 | -2.19 | 0.041 |
| 27 | PG(12:0/21:0) | 1.65 | 736.5261 | 13.68 | -1.81 | 0.009 |
| 28 | PC(18:4(6Z,9Z,12Z,15Z)/P-16:0) | 1.39 | 737.5389 | 14.61 | -1.27 | 0.039 |
| 29 | PA(20:2(11Z,14Z)/22:4(7Z,10Z,13Z,16Z)) | 1.34 | 776.5384 | 16.91 | -1.00 | 0.048 |
| 30 | PA(22:4(7Z,10Z,13Z,16Z)/22:6(4Z,7Z,10Z,13Z,16Z,19Z)) | 1.43 | 796.5074 | 13.47 | -3.41 | 0.032 |
| 31 | methyl 2-Pyrimidine Carboxylate | 1.63 | 138.0429 | 6.05 | 0.59 | 0.001 |
| 32 | 4-Pyridoxolactone | 1.55 | 165.0424 | 4.18 | 3.42 | 0.002 |
| 33 | (R)-3-Hydroxy-5-phenylpentanoic acid | 1.29 | 194.0940 | 5.51 | 0.84 | 0.022 |
| 34 | 4-Carboxyphenylglycine | 1.64 | 195.0527 | 3.85 | 3.64 | 0.001 |
| 35 | Metyrosine | 1.70 | 195.0892 | 6.53 | 0.82 | 0.000 |
| 36 | 3-(3,4-Dihydroxyphenyl)pyruvate | 1.59 | 196.0371 | 4.49 | -3.77 | 0.001 |
| 37 | 5-L-Glutamyl-taurine | 1.54 | 254.0572 | 4.28 | -4.98 | 0.003 |
| 38 | Homolanthionine | 1.41 | 268.0731 | 5.12 | -5.43 | 0.009 |
| 39 | D-Pantothenoyl-L-cysteine | 1.27 | 322.1201 | 5.77 | -9.17 | 0.025 |
| 40 | 2-Phenylaminoadenosine | 1.22 | 358.1407 | 4.12 | -5.29 | 0.033 |
| 41 | 1α,17α,21-trihydroxy-20-oxo-22,23,24,25,26,27-hexanorvitamin D3 | 1.23 | 362.2078 | 7.78 | -1.35 | 0.032 |
| 42 | 2-S-Glutathionyl acetate | 1.28 | 365.0891 | 5.25 | -4.79 | 0.024 |
| 43 | 2R-hydroxy-hexacosanoic acid | 1.40 | 412.3906 | 13.47 | 0.66 | 0.010 |
| 44 | Pelargonidin 3-rhamnoside | 1.41 | 416.1096 | 3.78 | -8.79 | 0.009 |
| 45 | Coumestrol 3-O-glucoside | 1.45 | 430.0889 | 3.90 | -8.30 | 0.007 |
| 46 | N-(3-pyridyl)-Indomethacin amide | 1.45 | 433.1207 | 3.84 | 4.43 | 0.006 |
| 47 | 9,10-dibromo-stearic acid | 1.20 | 440.0921 | 0.90 | 0.85 | 0.038 |
| 48 | 11α-ethyl-1α,25-dihydroxyvitamin D3 | 1.31 | 444.3595 | 15.34 | -2.45 | 0.019 |
| 49 | Se-Adenosylselenomethionine | 1.46 | 446.0838 | 3.86 | -7.88 | 0.006 |
| 50 | 1α,25-dihydroxy-24a,24b,24c-trihomovitamin D3 | 1.23 | 458.3749 | 16.19 | -4.98 | 0.032 |
| 51 | 1-O-alpha-D-glucopyranosyl-1,2-eicosandiol | 1.50 | 476.3697 | 10.19 | 1.50 | 0.004 |
| 52 | Sulindac sulfide glucuronide | 1.20 | 516.1253 | 3.85 | -7.08 | 0.037 |
| 53 | Cer(t18:0/24:0(2-OH)) | 1.45 | 683.6397 | 13.54 | 1.11 | 0.006 |

Note: VIP, variable importance in projection; CompMW, compound molecular weight; RT, retention time.

Table S3 Effect of +Mo treatment on significantly altered metabolites in the rhizosphere soil of maize and soybean crops

| No. | Metabolite | VIP | CompMW | RT | fold M +Mo/S +Mo | T.test |
| --- | --- | --- | --- | --- | --- | --- |
| 1 | 5,6-Dihydroxyindole-2-carboxylic acid | 1.58 | 193.0368 | 3.83 | 12.10 | 0.007 |
| 2 | GlcCer(d14:2(4E,6E)/20:0(2OH)) | 1.27 | 713.5407 | 13.77 | -1.16 | 0.043 |
| 3 | Sucrose | 1.58 | 342.1151 | 0.87 | -0.35 | 0.007 |
| 4 | 1α-hydroxy-25-methoxyvitamin D3 | 1.37 | 430.3432 | 14.52 | -0.85 | 0.026 |
| 5 | L-Valine | 1.36 | 117.0785 | 17.12 | -0.13 | 0.027 |
| 6 | Phenylacetic acid | 1.34 | 136.0521 | 4.86 | -1.05 | 0.031 |
| 7 | N-Acetyl-p-benzoquinonimine | 1.80 | 149.0472 | 3.63 | 10.39 | 0.001 |
| 8 | 4-Pyridoxolactone | 1.68 | 165.0421 | 4.18 | 10.45 | 0.003 |
| 9 | 5-Hydroxyindol-2-carboxylic acid | 1.78 | 177.0421 | 3.63 | 7.47 | 0.001 |
| 10 | Choline sulfate | 1.73 | 183.0561 | 0.87 | -0.96 | 0.002 |
| 11 | Phosphohydroxypyruvic acid | 1.33 | 183.9779 | 17.15 | -0.12 | 0.031 |
| 12 | Salicyluric acid | 1.62 | 195.0526 | 3.63 | 11.60 | 0.005 |
| 13 | 4-(2-Aminophenyl)-2,4-dioxobutanoic acid | 1.69 | 207.0527 | 3.63 | 11.45 | 0.002 |
| 14 | Urolithin B | 1.41 | 212.0468 | 4.63 | -10.19 | 0.021 |
| 15 | 4-(2-Amino-3-hydroxyphenyl)-2,4-dioxobutanoic acid | 1.55 | 223.0474 | 3.84 | 11.02 | 0.008 |
| 16 | 5-L-Glutamyl-taurine | 1.42 | 254.0561 | 4.28 | -7.55 | 0.019 |
| 17 | Adenosine | 1.55 | 267.0963 | 0.89 | -1.05 | 0.008 |
| 18 | Homolanthionine | 1.67 | 268.0729 | 5.12 | -4.23 | 0.003 |
| 19 | D-Pantothenoyl-L-cysteine | 1.35 | 322.1195 | 5.26 | -8.92 | 0.029 |
| 20 | 4,8 dimethylnonanoyl carnitine | 1.55 | 329.2556 | 6.11 | -0.14 | 0.008 |
| 21 | Glycidyl oleate | 1.66 | 338.2811 | 10.10 | -1.27 | 0.003 |
| 22 | PA(O-16:0/0:0) | 1.27 | 396.2650 | 4.82 | 1.17 | 0.044 |
| 23 | (5E)-vitamin D2 | 1.36 | 396.3377 | 15.93 | -0.70 | 0.026 |
| 24 | (17E)-1α,25-dihydroxy-17,20-didehydro-21-norvitamin D3 | 1.29 | 400.2965 | 6.21 | -0.95 | 0.038 |
| 25 | MG(0:0/22:1(13Z)/0:0) | 1.73 | 412.3544 | 10.19 | 1.98 | 0.002 |
| 26 | Vitamin D5 | 1.41 | 412.3691 | 10.04 | -0.94 | 0.020 |
| 27 | 1α-hydroxy-22-(3-methylphenyl)-23,24,25,26,27-pentanorvitamin D3 | 1.49 | 420.3017 | 10.56 | -2.24 | 0.013 |
| 28 | Glutinone | 1.26 | 424.3693 | 11.39 | -1.52 | 0.045 |
| 29 | Coumestrol 3-O-glucoside | 1.62 | 430.0884 | 3.90 | -13.81 | 0.005 |
| 30 | 26,27-Dihomo-1α-hydroxyvitamin D2 | 1.69 | 440.3638 | 8.97 | -5.84 | 0.002 |
| 31 | MG(0:0/24:1(15Z)/0:0) | 1.48 | 440.3851 | 11.50 | 0.78 | 0.013 |
| 32 | PA(O-20:0/0:0) | 2.00 | 454.3434 | 6.52 | -4.98 | 0.000 |
| 33 | 1α,24-Dihydroxy-22-ene-24-cyclopropylvitamin D3 | 1.71 | 454.3434 | 8.76 | -2.06 | 0.002 |
| 34 | 1α,25-dihydroxy-22,23-didehydro-24a,24b,24c-trihomovitamin D3 | 1.60 | 456.3591 | 8.53 | -5.55 | 0.005 |
| 35 | PA(20:0/0:0) | 1.33 | 466.3045 | 8.93 | -0.63 | 0.032 |
| 36 | (20R)-24-Hydroxy-19-norgeminivitamin D3 | 1.38 | 506.3946 | 13.57 | -1.55 | 0.025 |
| 37 | Cer(d14:2(4E,6E)/20:1(11Z)) | 1.87 | 533.4781 | 15.13 | -1.76 | 0.000 |
| 38 | β-Carotene | 1.55 | 536.4391 | 14.18 | -0.87 | 0.008 |
| 39 | 5-O-a-L-Arabinofuranosyl-L-arabinose | 1.57 | 564.1876 | 5.91 | -1.60 | 0.007 |
| 40 | Cer(t18:0/16:0(2OH)) | 1.27 | 571.5153 | 15.10 | -0.45 | 0.042 |
| 41 | Apigenin 7-glucuronide-4'-rhamnoside | 1.63 | 592.1407 | 3.53 | -3.90 | 0.004 |
| 42 | PI(18:4(6Z,9Z,12Z,15Z)/0:0) | 1.55 | 592.2663 | 10.14 | -1.27 | 0.008 |
| 43 | PA(16:0/16:0) | 1.30 | 648.4706 | 15.50 | -0.60 | 0.036 |
| 44 | PG(12:0/21:0) | 1.26 | 736.5261 | 13.68 | -1.12 | 0.045 |
| 45 | PC(18:4(6Z,9Z,12Z,15Z)/P-16:0) | 1.34 | 737.5389 | 14.61 | -0.72 | 0.031 |
| 46 | PA(18:1(9Z)/22:4(7Z,10Z,13Z,16Z)) | 1.37 | 750.5235 | 16.77 | -0.37 | 0.026 |
| 47 | PE(P-18:0/22:6(4Z,7Z,10Z,13Z,16Z,19Z)) | 1.48 | 775.5536 | 15.07 | -0.51 | 0.013 |
| 48 | Lactic acid | 1.18 | 90.0316 | 1.21 | 0.80 | 0.038 |
| 49 | N-Acetylleucine | 1.16 | 173.1050 | 3.57 | 1.81 | 0.042 |
| 50 | (E)-2-Butenyl-4-methyl-threonine | 1.22 | 187.1207 | 3.80 | 2.06 | 0.030 |
| 51 | 4-Carboxyphenylglycine | 1.58 | 195.0527 | 3.52 | 11.68 | 0.002 |
| 52 | Metyrosine | 1.27 | 195.0892 | 6.53 | 0.59 | 0.023 |
| 53 | 3-(3,4-Dihydroxyphenyl)pyruvate | 1.50 | 196.0371 | 4.49 | -3.71 | 0.004 |
| 54 | Phenanthrene-3,4-diol | 1.31 | 210.0681 | 4.28 | -9.08 | 0.017 |
| 55 | 5-L-Glutamyl-taurine | 1.54 | 254.0572 | 4.28 | -6.03 | 0.002 |
| 56 | Homolanthionine | 1.69 | 268.0731 | 5.12 | -3.28 | 0.000 |
| 57 | 9E-Octadecenedioic acid | 1.17 | 312.2293 | 6.36 | 1.89 | 0.040 |
| 58 | 9,10-dihydroxy-12-octadecenoic acid | 1.55 | 314.2451 | 6.56 | 0.62 | 0.002 |
| 59 | D-Pantothenoyl-L-cysteine | 1.25 | 322.1201 | 5.77 | -6.02 | 0.026 |
| 60 | 18-hydroxy-9S,10R-dihydroxy-stearic acid | 1.21 | 332.2556 | 6.06 | 1.41 | 0.032 |
| 61 | Sulindac sulfide | 1.62 | 340.0942 | 3.76 | 1.85 | 0.001 |
| 62 | Oxoglaucine | 1.15 | 351.1097 | 5.70 | -0.88 | 0.044 |
| 63 | S-Adenosylmethioninamine | 1.14 | 354.1491 | 9.79 | 2.40 | 0.048 |
| 64 | 2-glyceryl-PGD2 | 1.20 | 426.2638 | 11.43 | 1.39 | 0.034 |
| 65 | N-(3-pyridyl)-Indomethacin amide | 1.52 | 433.1207 | 3.84 | #DIV/0! | 0.003 |
| 66 | Se-Adenosylselenomethionine | 1.58 | 446.0838 | 3.86 | -9.11 | 0.002 |
| 67 | 1α,25-dihydroxy-22,23-didehydro-24a,24b,24c-trihomovitamin D3 | 1.31 | 456.3592 | 9.26 | -3.33 | 0.017 |
| 68 | 1α,25-dihydroxy-2β-(3-hydroxypropoxy)vitamin D3 | 1.66 | 490.3644 | 6.52 | -8.20 | 0.000 |
| 69 | Cer(d14:1(4E)/20:1(11Z)(2OH)) | 1.51 | 551.4897 | 15.14 | -1.03 | 0.003 |
| 70 | Cer(d15:2(4E,6E)/20:0(2OH)) | 1.18 | 565.5045 | 15.98 | -0.63 | 0.038 |

Note: VIP, variable importance in projection; CompMW, compound molecular weight; RT, retention time.
